# Hypercohones A–C, acylphloroglucinol derivatives with *homo*-adamantane cores from *Hypericum cohaerens*

**DOI:** 10.1007/s13659-013-0032-9

**Published:** 2013-09-06

**Authors:** Xia Liu, Xing-Wei Yang, Chao-Qun Chen, Chun-Yan Wu, Jing-Jing Zhang, Jun-Zeng Ma, Huan Wang, Qin-Shi Zhao, Li-Xin Yang, Gang Xu

**Affiliations:** 1 State Key Laboratory of Phytochemistry and Plant Resources in West China, Kunming Institute of Botany, Chinese Academy of Sciences, Kunming, 650201 China; 2 University of Chinese Academy of Sciences, Beijing, 100049 China

**Keywords:** *Hypericum cohaerens*, adamantane, acylphloroglucinol, hypercohone

## Abstract

Three new *homo*-adamantanyl type natural products were derived from polyprenylated polycyclic acylphloroglucinol. Hypercohones A-C (**1–3**), along with five other known hypercohones (**4–8**), were isolated from the aerial parts of *Hypericum cohaerens*. The structures of **1–3** were elucidated on the basis of comprehensive spectroscopic analysis. The inhibitory activities of these isolates against five human cancer cell lines *in vitro* were tested.

**Electronic Supplementary Material:**

Supplementary material is available for this article at 10.1007/s13659-013-0032-9 and is accessible for authorized users.

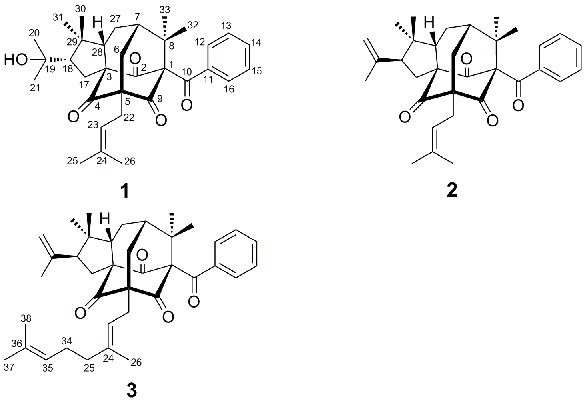

## Introduction

Adamantanes, featured with a "diamond-like" caged core, have been of interest to medicinal chemists since the early 1960s, when they were first used clinically for the treatment of influenza in the form of amantadine.[1,2] In the past 50 years, adamantanes have yielded many compounds directed toward many different drug targets. Based on our detailed literature research, the adamantyl group was present in seven registered therapeutic compounds used currently for clinical use, and in many more compounds that were currently being developed as potential agents for the treatment of conditions such as iron overload disease, cancer, malaria and tuberculosis.[3,4] In many cases, the adamantyl group has been found to increase drug-like qualities of a lead compound without increasing toxicity. The value of the adamantyl group in drug design is multi-dimensional. From a medicinal chemist's point of view, the adamantyl group can be used either as a scaffold for the development of therapeutic agents, (i.e. memantine and amantadine), or as a modifier of the pharmacokinetics of a compound.[1–4]

It was once assumed that living organisms could not synthesize adamantane derivatives, whose synthesis was considered purely abiotic. This view was finally refuted in 1996 when pukenetione A, the first adamantane derivative isolated and identified from the plant *Clusia plukenetii* (Guttiferae).[5,6] To date, less than 50 natural products with adamantane core skeletons have been reported. All of these compounds were characterized from the Guttiferae (Clusiaceae) family of plants by the further cyclization of polyprenylated

*To whom correspondence should be addressed. E-mail: xugang008@mail.kib.ac.cn

**Figure 1.** Plausible biogenetic pathway of adamantane and *homo*-adamantane type metabolites polycyclic acyphloroglucinols (PPAPs).[7–9] Natural adamantanes can be divided into two types based on their structural characteristics; adamantane and *homo*-adamantane. The two types of metabolites were both derived from polyprenylated polycyclic acyphloroglucinols by the further cyclizations of C-3 to the different position of prenyl groups at C-7 (Figure 1).[10–12]

The genus *Hypericum,* occurrs commonly in temperate regions throughout the world, and has been a key plant used in traditional medical systems in many countries.[13] *H. cohaerens* N. Robson is an endemic plant distributed in Guizhou and Yunnan provinces of China.[14] Previous phytochemical investigation of this plant led to the isolation of hypercohin A, a polyprenylated acylphloroglucinol derivative featuring with an unusual bicyclo[5.3.1]-hendecane core.[15] With the aim of searching for bioactive and structural interesting acylphloroglucinol derivatives and continuing our systematic phytochemical studies of *Hypericum spp.* plants, the chemical constituents of this plant were investigated. In total, three new adamantyl type metabolites (hypercohones A–C, **1**–**3**) were isolated, with an additional five known metabolites. Herein, this report describes the isolation and structural elucidation of these adamantyl type natural products from *H. cohaerens*.

## Results and Discussion

The MeOH extract of the air-dried and powdered aerial parts of *H. cohaerens* (10.0 kg) was subjected to a silica gel column to afford five fractions A–E. Fraction B was subjected to a series of chromatographic methods, and led to the isolation of three new adamantyl acylphloroglucinol derivatives, namely hypercohones A–C (**1**–**3**), along with five known analogues, sampsonione D (**4**),[16] sampsonione G (**5**),[16] plukenetione B (**6**),[17] hypersampsone I (**7**),[18] and sampsonione H (**8**).[16]

Hypercohone A (**1**) was obtained as a colorless oil. Its molecular formula $C_{33}H_{42}O_5$ was established by the positive HREIMS (*m/z* 518.3022, $[M]^+$, calcd 518.3032), indicating 13 degrees of unsaturation. The IR spectrum showed obvious absorption bands for carbonyl (1737 and 1703 $cm^{-1}$) groups. Careful analysis of $^{13}C$ and DEPT NMR data (Table 1) indicated that the characteristic signals for a benzoyl substituted adamantyl metabolite could be easily distinguished as below: three non-conjugated carbonyl ($\delta_C$ 204.9, C-2; $\delta_C$ 204.6, C-4; and $\delta_C$ 206.6, C-9), four quaternary carbons at $\delta_C$ 82.7 (C-1), 72.2 (C-3), 69.0 (C-5), and 49.0 (C-8), two methine at $\delta_C$ 43.4 (C-7) and 57.3 (C-28), one methylene at $\delta_C$ 36.3 (C-6), and a benzoyl group ($\delta_C$ 194.3, C-10; $\delta_C$ 136.5, C-11; $\delta_C$ 129.6, C-12 and C-16; $\delta_C$ 129.3, C-13 and C-15; and $\delta_C$ 133.4, C-14).[5–12] Obvious signals for an olefinic proton of isoprenyl group ($\delta_H$ 5.27, t, $J = 7.5$ Hz), eight methyls ($\delta_H$ 0.97 –1.73, s), and a mono-substituted benzene group ($\delta_H$ 7.01, 2H, d, $J = 8.3$ Hz, H-12 and H-16; $\delta_H$ 7.31, 2H, dd, $J = 8.3$, 7.5 Hz, H-13 and H-15; $\delta_H$ 7.43, 1H, t, $J = 7.5$ Hz, H-14) could also be found in the $^1H$ NMR spectrum (Table 2). These observations, conjugated with the fact that almost all of the natural adamantyl compounds were isolated from the plants of Guttiferae family, indicated that **1** could be ascribed to be adamantyl or *homo*-adamantyl type metabolites. The diagnostic signal found in the $^{13}C$ NMR spectrum at $\delta_C$ 23.6 (C-27) for a typical *homo*-adamantyl type acylphloroglucinol suggested that **1** was a natural product with a *homo*-adamantane skeleton.[10,11,16–18]

The $^1H$ and $^{13}C$ NMR spectral data of **1** were very similar to those of sampsonione G (**5**),[16] indicating these two compounds shared a same planar structure. Biogenetically, the benzoyl group was usually located at C-1, which could be further evidenced by the chemical shift of C-1 ($\delta_C$ 82.7, s), otherwise the chemical shift might be in the upfield region. In the HMBC spectrum, the correlations from both Me-32 and Me-33 to C-1, C-7, and C-8, from both Me-30 and Me-31 to C-18, C-28, and C-29, from Me-20 and Me-21 to C-18, and C-19, from Me-25 and Me-26 to C-23, and C-24, from $H_2$-22 to C-4, C-5, C-6, and C-9, from H-6 to C-4, C-5, C-9, and C-27, as well as from $H_2$-17 to C-2, C-3, C-4, C-19, and C-29 can all be found (Figure 2). These HMBC correlations, coupled with the proton spin systems $H_2$-6/H-7/$H_2$-27/H-28, $H_2$-17/H-18, $H_2$-22/H-23,

**Figure 2.** Key HMBC(→), $^1H$-$^1H$ COSY(▬), and ROESY (↔) correlations of **1**

**Table 1. $^{13}$C NMR data for compounds 1–3 ($\delta$ in ppm, 150 MHz)**

| position | 1[a] | 2[b] | 3[b] | position | 1[a] | 2[b] | 3[b] |
|---|---|---|---|---|---|---|---|
| 1 | 82.7 C | 82.7 C | 82.7 C | 21 | 30.0 $CH_3$ | 23.7 $CH_3$ | 23.7 $CH_3$ |
| 2 | 204.9 C | 205.6 C | 205.6 C | 22 | 30.3 $CH_2$ | 30.3 $CH_2$ | 30.3 $CH_2$ |
| 3 | 72.2 C | 73.0 C | 73.0 C | 23 | 120.3 CH | 120.0 CH | 120.1 CH |
| 4 | 204.6 C | 203.8 C | 203.8 C | 24 | 136.1 CH | 135.4 C | 139.2 CH |
| 5 | 69.0 C | 68.5 C | 68.6 C | 25 | 26.3 $CH_3$ | 26.2 $CH_3$ | 40.7 $CH_2$ |
| 6 | 36.3 $CH_2$ | 35.3 $CH_2$ | 35.2 $CH_2$ | 26 | 18.2 $CH_3$ | 18.0 $CH_3$ | 16.3 $CH_3$ |
| 7 | 43.4 CH | 42.9 CH | 42.9 CH | 27 | 23.6 $CH_2$ | 26.6 $CH_2$ | 26.6 $CH_2$ |
| 8 | 49.0 C | 48.0 C | 48.0 C | 28 | 57.3 CH | 55.1 CH | 55.1 CH |
| 9 | 206.6 C | 205.8 C | 205.7 C | 29 | 47.5 C | 45.1 C | 45.1 C |
| 10 | 194.3 C | 193.8 C | 193.8 C | 30 | 29.5 $CH_3$ | 28.0 $CH_3$ | 28.0 $CH_3$ |
| 11 | 136.5 C | 136.0 C | 136.0 C | 31 | 17.4 $CH_3$ | 26.0 $CH_3$ | 25.9 $CH_3$ |
| 12(16) | 129.6 CH | 129.1 CH | 129.1 CH | 32 | 25.6 $CH_3$ | 25.2 $CH_3$ | 25.3 $CH_3$ |
| 13(15) | 129.3 CH | 129.1 CH | 129.1 CH | 33 | 22.8 $CH_3$ | 22.6 $CH_3$ | 22.6 $CH_3$ |
| 14 | 133.4 CH | 133.2 CH | 133.2 CH | 34 | | | 27.1 $CH_2$ |
| 17 | 31.5 $CH_2$ | 34.4 $CH_2$ | 34.4 $CH_2$ | 35 | | | 124.9 CH |
| 18 | 60.8 CH | 56.7 CH | 56.7 CH | 36 | | | 131.7 C |
| 19 | 73.3 C | 145.5 C | 145.5 C | 37 | | | 26.0 $CH_3$ |
| 20 | 30.8 $CH_3$ | 113.2 $CH_2$ | 113.2 $CH_2$ | 38 | | | 17.7 $CH_3$ |

[a]Recorded in methanol-$d_4$; [b]Recorded in acetone-$d_6$.

and H-12/H-13/H-14/H-15/H-16 presented in the $^1$H-$^1$H COSY spectrum (Figure 2), confirmed that **1** possessed a same planar structure with sampsonione G.[16]

While side-by-side analysis of their NMR spectral data showed that the chemical shifts of C-17 ($\delta_C$ 31.5, t), C-27 ($\delta_C$ 23.6, t), and C-31 ($\delta_C$ 17.4, q) of **1** were downfield shifted obviously in the $^{13}$C NMR spectrum compared to the corresponding signals of **5** (C-17, $\delta_C$ 33.1, t; C-27, $\delta_C$ 26.7, t; and C-31, $\delta_C$ 28.2, q).

These observations indicated that **1** might be the stereoisomer of **5**. In the ROESY spectrum of **5**, diagnostic cross peaks of H-28/H-6b, H-28/H-30, and H-6a/Me-32 suggested the $\beta$-orientations of Me-30, Me-32, and H-28. All the ROESY correlations discussed above were almost identical with these of **5**, which suggested the same configuration of **1** and **5** except for the stereochemistry of C-18. Then, H-18 was deduced to be $\beta$-oriented (Figure 2) by the ROESY correlations of H-18/H-28 and H-18/Me-30. Therefore, the structure of **1** was elucidated and named hypercohone A.

Hypercohone B (**2**) was isolated as colorless oil and yielded a pseudomolecular ion peak at *m/z* 523.2811 ($[M + Na]^+$, calcd 523.2824) in the positive HRESIMS, indicative of the molecular formula $C_{33}H_{40}O_4$. Comparison of their 1D and 2D NMR data indicated that the structures of **1** and **2** were very similar to each other (Tables 1 and 2). The difference lied in that the oxygenated quaternary carbon (C-19, $\delta_C$ 73.3, s) and Me-20 ($\delta_C$ 30.8 q) in **1** were replaced by a double bond ($\delta_C$ 113.2, t, C-20; and 145.5, s, C-19) in **2**, which could be confirmed by HMBC correlations from H-20 ($\delta_H$ 4.89 and 4.76, each 1H, br. s) to C-18 ($\delta_C$ 56.7, d), C-19, and C-21 ($\delta_C$ 23.7, q) and from Me-21 ($\delta_H$ 1.77, s) to C-18, C-19 and C-20. The ROESY correlations of H-28/Me-30, H-28/H-6b, and H-6a/Me-32 showed that H-28, Me-30 and Me-32 were all $\beta$-oriented. Then H-18 was elucidated to be in the $\alpha$-orientation by its ROESY correlations with Me-31 (Figure 3). Thus, the structure of **2** was established as shown.

**Figure 3** Key ROESY correlations of **2**

Hypercohone C (**3**) had a molecular formula of $C_{38}H_{48}O_4$ from the HRESIMS, 68 mass units more than **2**, in accordance with the presence of an additional isoprenyl group. The 1D NMR data of **3** were similar to those of **2**, with the only difference being the presence of five more signals ascribable for an isoprenyl group at $\delta_C$ 27.1 (t, C-34), 124.9 (d, C-35), 131.7 (s, C-36), 26.0 (q, C-37), and 17.7 (q, C-38). This isoprenyl group was ascribed to connect to C-25 by the HMBC correlations of Me-26 to C-24 and C-25, of both Me-37and Me-38 to C-35 and C-36, coupled with the correlations of $H_2$-25/$H_2$-34 and $H_2$-34/H-35 observed in the $^1$H-$^1$H COSY spectrum. The ROESY correlations of H-28/Me-30, H-28/H-6b, H-6a/Me-32, and H-18/Me-31 showed that **3** had the same relative configurations as **2**. Accordingly, the structure of **3** was established and named hypercohone C.

The AChE inhibitory activity of all the new and known compounds was assayed using the method developed by Ellman et al.[19] None of these isolates showed any activities at the concentration of 50 μM. In addition, the eight isolates were

**Table 2.** $^1$**H NMR data for compounds 1–3 (600 MHz, *δ* in ppm, *J* in Hz)**

| pos. | 1[a] | 2[b] | 3[b] |
|---|---|---|---|
| 6a | 2.46 m | 2.51 m | 2.53 overlap |
| 6b | 2.34 d (13.2) | 2.44 m | 2.43 m |
| 7 | 2.17 m | 2.24 m | 2.24 m |
| 12(16) | 7.01 d (8.3) | 7.10 d (8.3) | 7.10 d (8.3) |
| 13(15) | 7.31 dd (8.3, 7.5) | 7.38 dd (8.3, 7.5) | 7.38 dd (8.3, 7.2) |
| 14 | 7.43 d (7.5) | 7.48 t (7.5) | 7.48 t (7.2) |
| 17 | 2.52 overlap | 2.86 overlap | 2.88 overlap |
| | 2.50 overlap | 1.68 overlap | 1.69 dd (13.2, 6.6) |
| 18 | 1.82 dd (13.2, 7.2) | 3.00 dd (12.6, 6.0) | 3.01 dd (12.6, 6.6) |
| 20 | 1.28 s | 4.89 br. s | 4.89 br. s |
| | | 4.76 br. s | 4.76 br. s |
| 21 | 1.32 s | 1.77 s | 1.77 s |
| 22 | 2.57 dd (15.3, 7.5) | 2.59 m | 2.61 dd (15.0, 7.5) |
| | 2.50 overlap | 2.54 m | 2.55 overlap |
| 23 | 5.27 t (7.5) | 5.27 t (7.5) | 5.30 t (7.5) |
| 25 | 1.73 s | 1.70 s | 2.02 overlap |
| 26 | 1.68 s | 1.66 s | 1.67s |
| 27 | 1.91 dd (13.9, 12.6) | 2.04 overlap | 2.05 overlap |
| | 1.76 m | 1.95 m | 1.95 m |
| 28 | 1.98 dd (12.6, 6.0) | 2.29 dd (12.6, 7.8) | 2.28 dd (12.3, 7.5) |
| 30 | 1.11 s | 0.85 s | 0.85 s |
| 31 | 0.97 s | 1.02 s | 1.02 s |
| 32 | 1.37 s | 1.38 s | 1.40 s |
| 33 | 1.34 s | 1.36 s | 1.36 s |
| 34 | | | 2.08 m |
| 35 | | | 5.08 t (6.9) |
| 37 | | | 1.64 s |
| 38 | | | 1.57 s |

[a]Recorded in methanol-$d_4$; [b]Recorded in acetone-$d_6$.

also tested for their cytotoxic effects against five human cancer cell lines, HL-60, A-549, SMMC-7721, MCF-7, and SW480, using the MTT method described previously.[20] All the tested samples showed no activities against the mentioned cell lines with $IC_{50} > 40$ μM.

## Experimental Section

**General Experimental Procedures.** Optical rotations were measured on a JASCO P-1020 polarimeter. UV spectra were detected on a Shamashim UV 2401 spectrometer. IR spectra were determined on a Broker Tensor-27 infrared spectrophotometer with Br disks. 1D and 2D NMR spectra were recorded on DRX-600 spectrometers using TMS as an internal standard. Unless otherwise specified, chemical shifts (*δ*) were expressed in ppm with reference to the solvent signals. ESIMS analysis and HREIMS were determined carried out on a Waters Auto spec Premier P776 mass spectrometer. Semi-preparative HPLC was performed on an Agile 1100 HPLC with a Razorback SB-$C_{18}$ (9.4 mm × 25 cm) column. Silica gel (100–200 and 200–300 mesh, Qingdao Marine Chemical Co., Ltd., Qingdao, China), and Amphichroic RP-18 gel (40–63 μm, Merck, Darmstadt, Germany) and MCI gel (75–150 μm, Mitsubishi Chemical Corporation, Tokyo, Japan) were used for column chromatography.

**Plant Material.** The aerial parts of *H. cohaerens* N. Robson were collected in Daguan prefecture, Yunnan Province, China, in October 2009. The plant was identified by Dr. En-De Liu, Kunming Institute of Botany, Kunming, China. A voucher specimen was deposited with Kunming Institute of Botany with identification number 200910H01.

**Extraction and Isolation.** The aerial parts of *H. cohaerens* (10.0 kg) were powdered and percolated with MeOH at room temperature and filtered. The filtrate was evaporated *in vacuo* to be concentrated. The obtained crude extract was subjected to a silica gel column chromatography eluted with petroleum ether-$Me_2CO$ in gradient to produce five fractions; A–E. Fraction B (86.4 g) was separated over a MCI-gel column (MeOH-$H_2O$ from 8:2 to 10:0) to obtain five fractions (B1–B5). Fr. B1 (18.5 g) was then chromatographed on a silica gel column, eluted with petroleum ether-$Me_2CO$ (from 50:1 to 10:1), to yield four fractions (B1a–B1d). Fr. B1a (9.3 g) was repeatedly subjected to silica gel columns, eluted with petroleum ether-EtOAc (from 50:1 to 6:1) and then was further purified by semi-preparative HPLC (MeOH-$H_2O$, 95:5) to afford **2** (4 mg), **3** (4 mg), sampsonione D (**4**, 2 mg), hypersampsone I (**7**, 8 mg) and sampsonione H (**8**, 3 mg). Fr. B3 was separated over a MCI-gel column (MeOH-$H_2O$ from 85:15 to 100:0) to obtain five fractions (Fr. B3a–B3e). Fr. B3b was then chromatographed on a silica gel column, eluted with petroleum ether-$Me_2CO$ (from 9:1 to 7:3), to yield seven fractions (Fr. B3b1–B3b7). Compounds **1** (20 mg), Plukenetione B (**6**, 20 mg) and sampsonione G (**5**, 13 mg) were isolated from Fr. B3b3 (200 mg) by chromatographed on $C_{18}$ silica gel columns, silica gel columns and repeated semi-preparative HPLC.

**Hypercohone A (1):** colorless oil; $[\alpha]_D^{22}$ + 3.87 (*c* 0.21, MeOH); UV (MeOH) $\lambda_{max}$ ( log *ε*) 274 (3.7), 224 (4.5), 202 (4.8) nm; IR (Br) $\nu_{max}$ 3446, 2974, 2926, 2874, 1728, 1667, 1621, 1468, 1368 $cm^{-1}$; $^1$H and $^{13}$C NMR data, see Tables 1 and 2; positive ESIMS *m/z* 541 $[M + Na]^+$; HREIMS *m/z* 518.3022 (calcd for $C_{33}H_{42}O_5$ $[M]^+$, 518.3032).

**Hypercohone B (2):** colorless oil; $[\alpha]_D^{24}$ – 37.2 (*c* 0.11, MeOH); UV (MeOH) $\lambda_{max}$ (log *ε*) 245 (4.1) and 207 (4.2) nm; IR (KBr) $\nu_{max}$ 2962, 2925, 1736, 1703, 1686, 1637, 1448, 1390 and 1236 $cm^{-1}$; $^1$H and $^{13}$C NMR data, see Tables 1 and 2; positive ESIMS *m/z* 523 $[M + Na]^+$; HRESIMS *m/z* 523.2811 (calcd for $C_{33}H_{40}O_4Na$ $[M + Na]^+$, 523.2824 ).

**Hypercohone C (3):** colorless oil; $[\alpha]_D^{24}$ – 38.0 (*c* 0.09, MeOH); UV (MeOH) $\lambda_{max}$ ( log *ε*) 245 (4.2) and 207 (4.4) nm; IR (KBr) $\nu_{max}$ 2962, 2926, 1736, 1703, 1686, 1448, 1390 and 1235 $cm^{-1}$; $^1$H and $^{13}$C NMR data, see Tables 1 and 2; positive ESIMS *m/z* 591 $[M + Na]^+$; HRESIMS *m/z* 591.3446 (calcd for $C_{38}H_{48}O_4Na$ $[M + Na]^+$, 591.3450 ).

**Acetylcholinesterase Inhibitory Activity.** Acetylcholinesterase inhibitory activity of the compounds was assayed by the spectrophotometric method developed by Ellman et al. Acetylthiocholine iodide (Sigma) was used as substrate in the assay. Compounds were dissolved in DMSO. The reaction mixture contained 1100 μL of phosphate buffer (pH 8.0), 10 μL of test compound solution (50 μM), and 40 μL of acetyl cholinesterase solution (0.04 U/100 μL), and the mixture was incubated for 20 min (30 °C). The reaction was initiated by the addition of 20 μL of DTNB (6.25 mM) and 20 μL of acetylthiocholine. The hydrolysis of acetylthiocholine was monitored at 405 nm after 30 min. Tacrine was used as positive control. All the reactions were performed in triplicate. The percentage inhibition was calculated as follows: % inhibition ) $(E - S)/E \times 100$ ($E$ is the activity of the enzyme without test compound and $S$ the activity of enzyme with test compound).

**Cytotoxcity Assays.** The following human tumor cell lines were used: HL-60, SMMC-7721, A-549, MCF-7, and SW-480, which were obtained from ATCC (Manassas, VA, USA). All cells were cultured in RPMI-1640 or DMEM medium (Hyclone, Logan, UT, USA), supplemented with 10% fetal bovine serum (FBS, Hyclone) at 37 °C in a humidified atmosphere with 5% $CO_2$. Cell viability was assessed by conducting colorimetric measurements of the amount of insoluble formazan formed in living cells based on the reduction of 3-(4,5-dimethylthiazol-2-yl)-2,5-diphenyltetrazolium bromide (MTT) (Sigma, St. Louis, MO, USA).17 Briefly, 100 $\mu$L of adherent cells was seeded into each well of a 96-well cell culture plate and allowed to adhere for 12 h before test compound addition, while suspended cells were seeded just before test compound addition, both with an initial density of 1 × 105 cells/mL in 100 $\mu$L of medium. Each tumor cell line was exposed to the test compound at various concentrations in triplicate for 48 h, with *cis*-platin and paclitaxel (Sigma) as positive control. After the incubation, MTT (100 $\mu$g) was added to each well, and the incubation continued for 4 h at 37 °C. The cells were lysed with 100 $\mu$L of 20% SDS−50% DMF after removal of 100 $\mu$L of medium. The optical density of the lysate was measured at 595 nm in a 96-well microtiter plate reader (Bio-Rad 680). The $IC_{50}$ value of each compound was calculated by Reed and Muench's method.

## Electronic Supplementary Material

Supplementary material is available in the online version of this article at http://dx.doi.org/10.1007/s13659-013-0032-9 and is accessible for authorized users.

## Acknowledgments

The work was financially supported by the foundations from NSFC (20972167), the Young Academic Leader Raising Foundation of Yunnan Province (No. 2009CI073), and the foundation from CAS to Dr Gang Xu.

**Open Access** This article is distributed under the terms of the Creative Commons Attribution License which permits any use, distribution, and reproduction in any medium, provided the original author(s) and source are credited.

## Electronic supplementary material

Supplementary material, approximately 1.91 MB.
