# Supplementary material for: Hypercohones A–C, acylphloroglucinol derivatives with homo-adamantane cores from Hypericum cohaerens
Source: Nat Prod Bioprospect. 2013 Sep 6;3(5):233–7. doi: 10.1007/s13659-013-0032-9 (PMC4131619; doi:10.1007/s13659-013-0032-9)
Supplement: Supplementary file 1 — Supplementary material, approximately 1.91 MB. [file 13659_2013_32_MOESM1_ESM.pdf]

Electronic Supplementary Material

Hypercohones A–C, acylphloroglucinol derivatives with *homo*-adamantane cores from *Hypericum cohaerens*

Xia LIU,[a,b] Xing-Wei YANG,[a,b] Chao-Qun CHEN,[a] Chun-Yan WU,[a] Jing-Jing ZHANG,[a,b] Jun-Zeng MA,[a] Huan WANG,[a] Qin-Shi ZHAO,[a] Li-Xin YANG,[a] and Gang XU[a,]*

[a]State Key Laboratory of Phytochemistry and Plant Resources in West China, Kunming Institute of Botany, Chinese Academy of Sciences, Kunming 650201, China

[b]University of Chinese Academy of Sciences, Beijing 100049, China

Received 5 April 2013; Accepted 16 April 2013

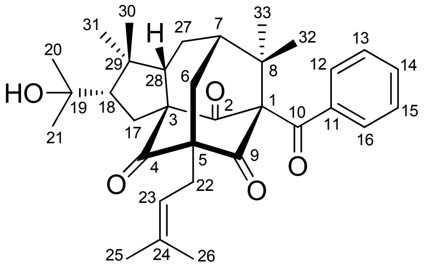

1

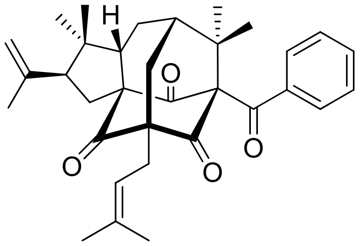

2

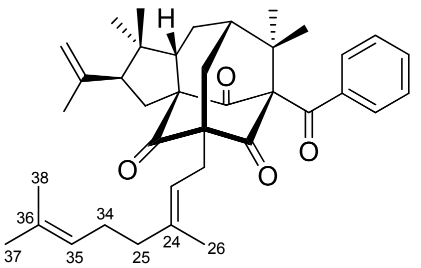

3

Structures of compounds **1**–**3**

*To whom correspondence should be addressed. E-mail: xugang008@mail.kib.ac.cn

S1. ^{1}H NMR spectrum of **1** (methanol-*d_4*, 600 MHz)
S2. ^{13}C NMR spectrum of **1** (methanol-*d_4*, 150 MHz)
S3. HSQC spectrum of **1**
S4. HMBC spectrum of **1**
S5. ^{1}H-^{1}H COSY spectrum of **1**
S6. ROESY spectrum of **1**
S7. ESI MS spectrum of **1**
S8. HREI MS spectrum of **1**
S9. IR (KBr disk) spectrum of **1**
S10. UV spectrum of **1** in MeOH
S11. ^{1}H NMR spectrum of **2** (acetone-*d_6*, 600 MHz)
S12. ^{13}C NMR spectrum of **2** (acetone-*d_6*, 150 MHz)
S13. HSQC spectrum of **2**
S14. HMBC spectrum of **2**
S15. ^{1}H-^{1}H COSY spectrum of **2**
S16. ROESY spectrum of **2**
S17. ESI MS spectrum of **2**
S18. HREI MS spectrum of **2**
S19. IR (KBr disk) spectrum of **2**
S20. UV spectrum of **2** in MeOH
S21. ^{1}H NMR spectrum of **3** (acetone-*d_6*, 600 MHz)
S22. ^{13}C NMR spectrum of **3** (acetone-*d_6*, 150 MHz)
S23. HSQC spectrum of **3**
S24. HMBC spectrum of **2**
S25. ^{1}H-^{1}H COSY spectrum of **3**
S26. ROESY spectrum of **3**
S27. ESI MS spectrum of **3**
S18. HREI MS spectrum of **3**
S19. IR (KBr disk) spectrum of **3**
S20. UV spectrum of **3** in MeOH

S1. ^{1}H NMR spectrum of **1** in methanol-d_4

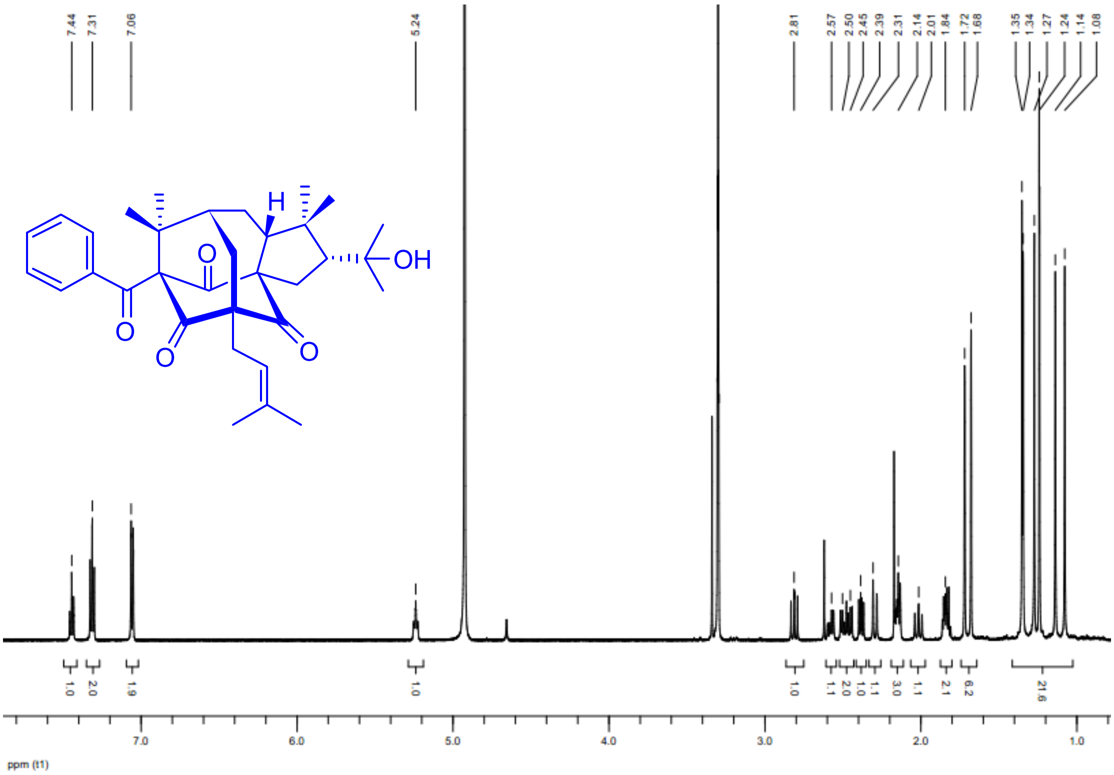

S2. ^{13}C NMR spectrum of **1** (methanol-$d_{4,}$ 150 MHz)

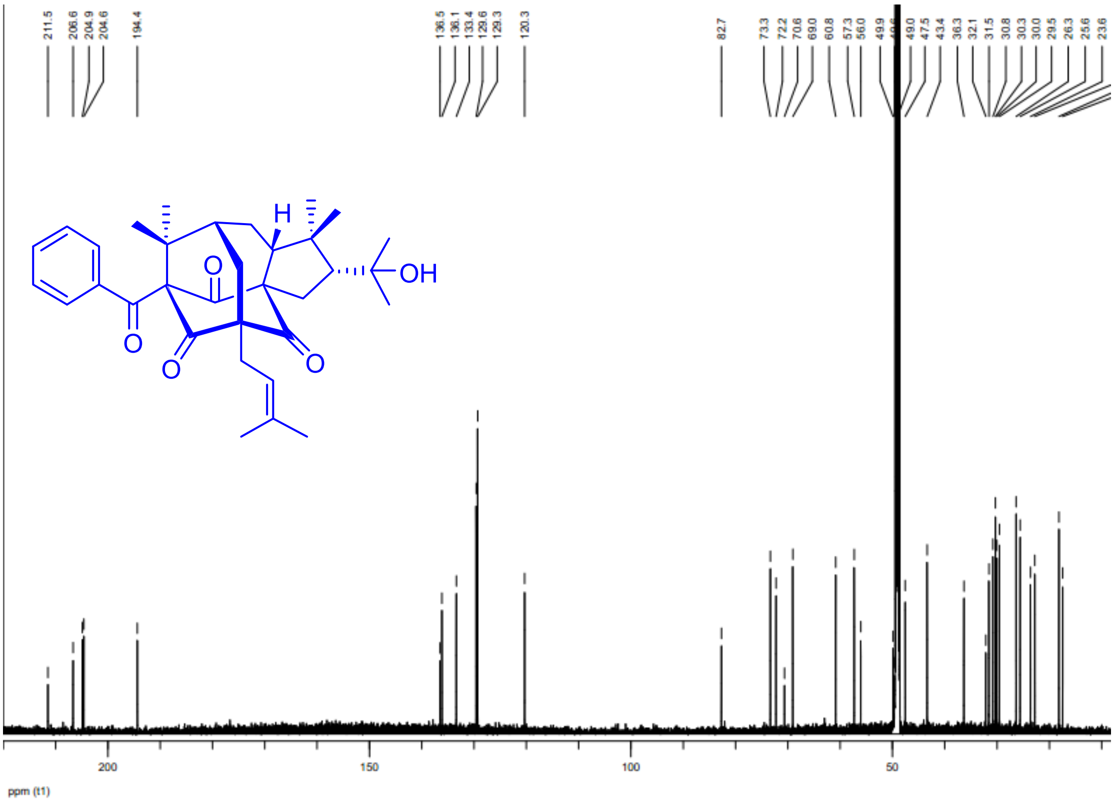

S3. HSQC spectrum of **1**

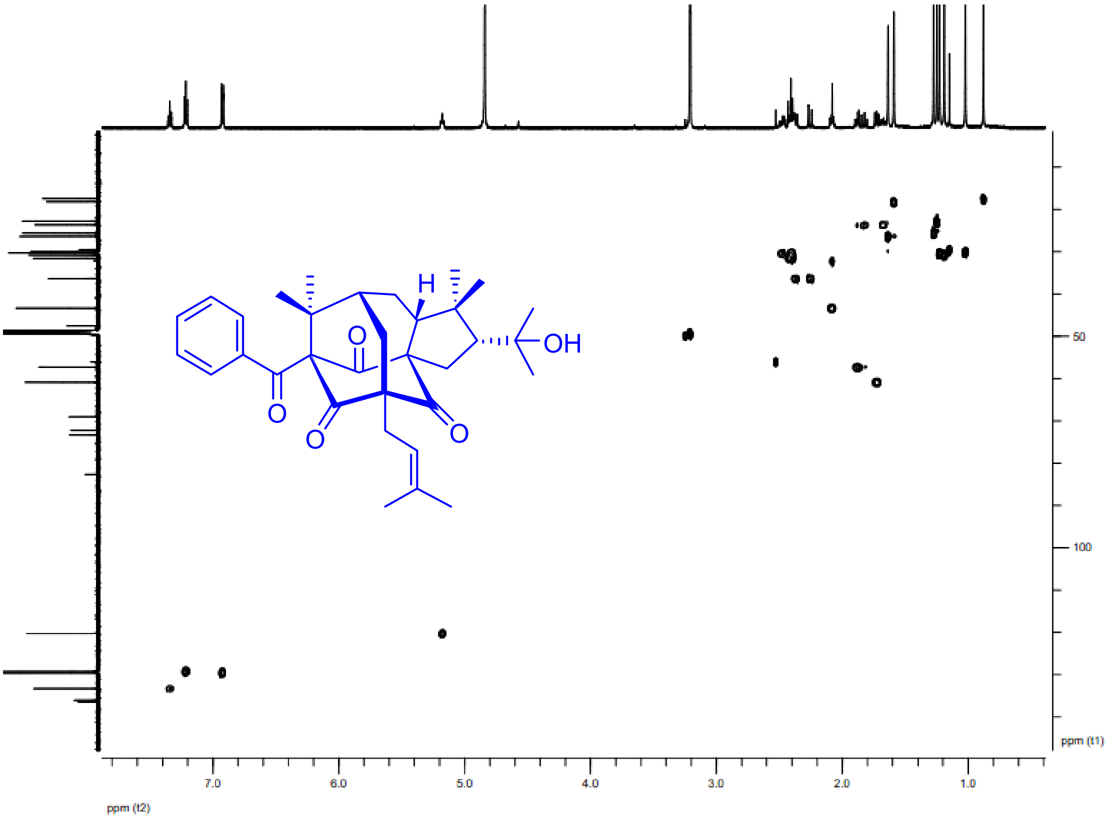

S4. HMBC spectrum of **1**

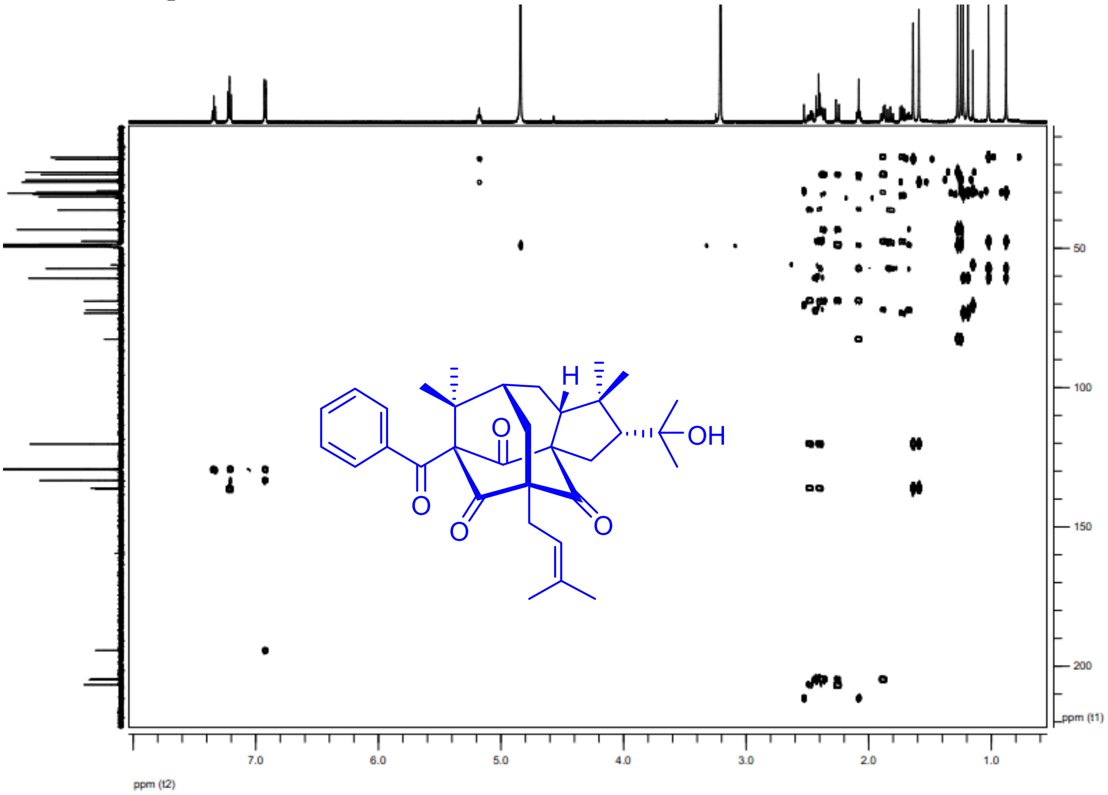

S5. ^{1}H-^{1}H COSY spectrum of **1**

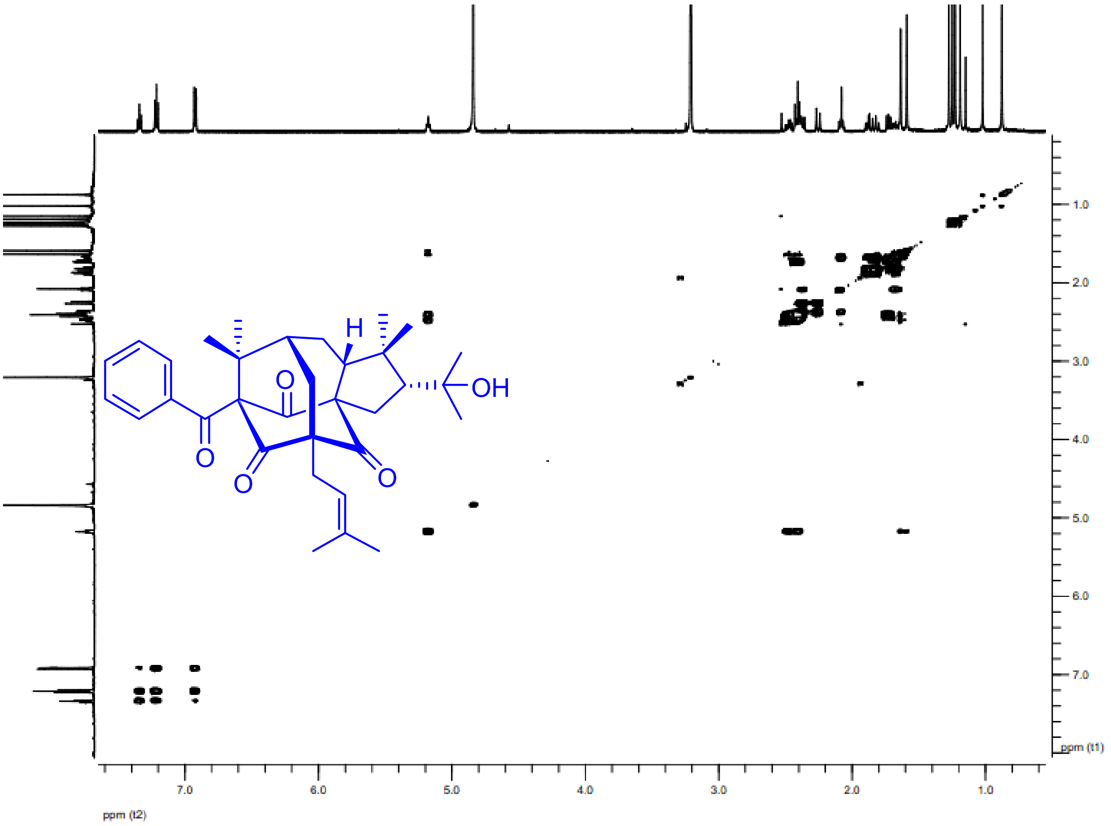

S6. ROESY spectrum of **1**

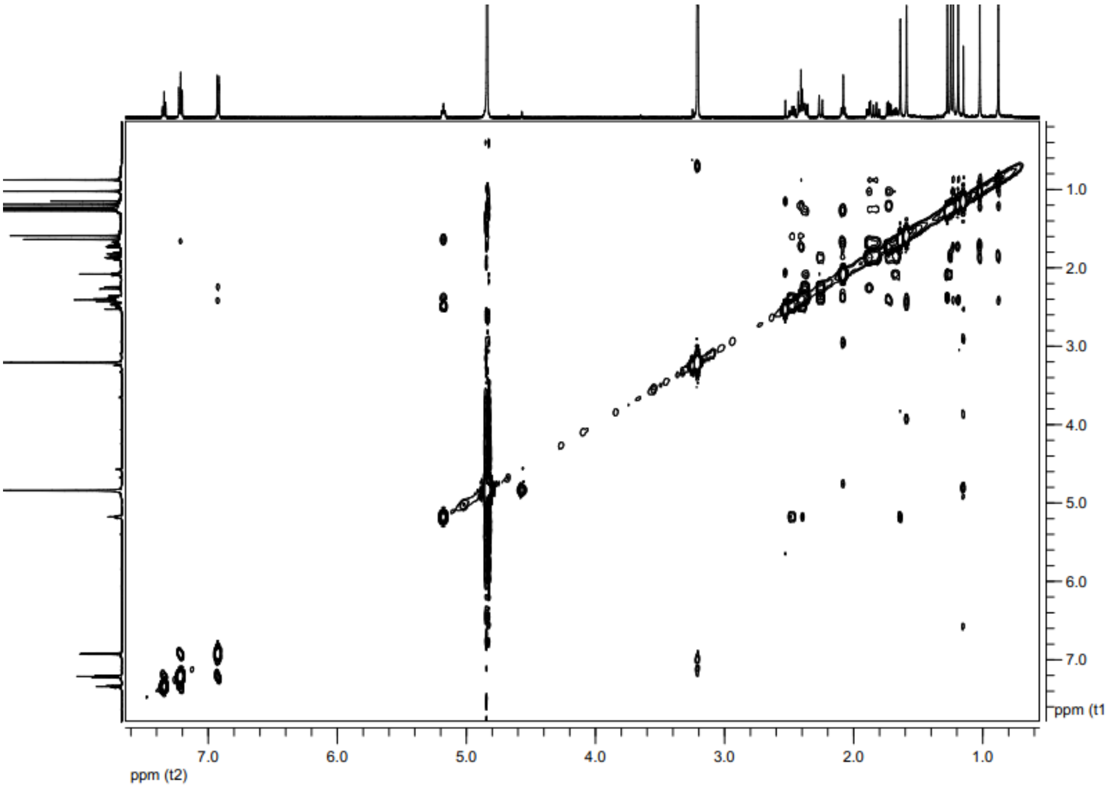

S7. ESI MS spectrum of **1**

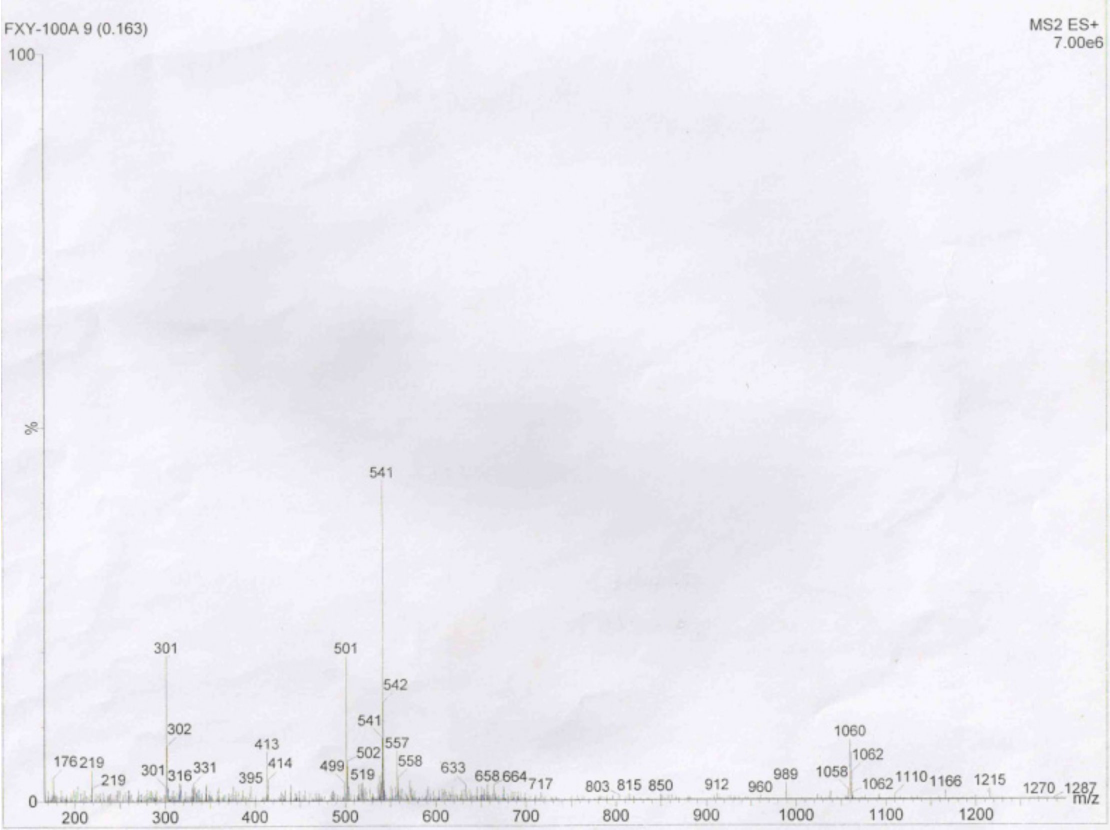

S8. HREI MS spectrum of **1**

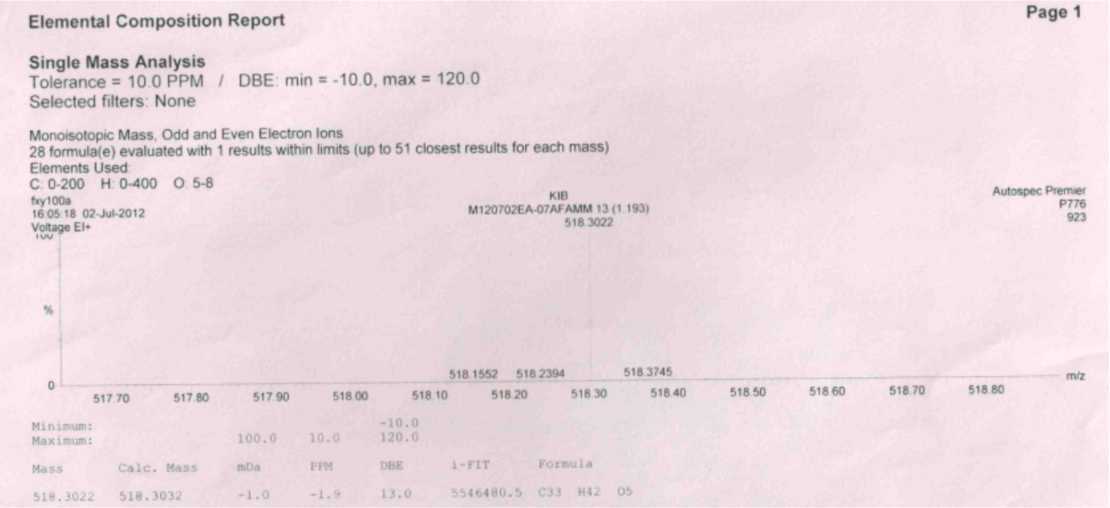
Elemental Composition Report

Page 1

Single Mass Analysis
Tolerance = 10.0 PPM / DBE: min = -10.0, max = 120.0
Selected filters: None

Monoisotopic Mass, Odd and Even Electron Ions
28 formula(e) evaluated with 1 results within limits (up to 51 closest results for each mass)
Elements Used:
C: 0-200 H: 0-400 O: 5-8

	Mass	Calc. Mass	mDa	PPM	DBE	i-FIT	Formula
Minimum:					-10.0		
Maximum:			100.0	10.0	120.0		
	518.3022	518.3032	-1.0	-1.9	13.0	5546480.5	C33 H42 O5

S9. IR (KBr disk) spectrum of **1**

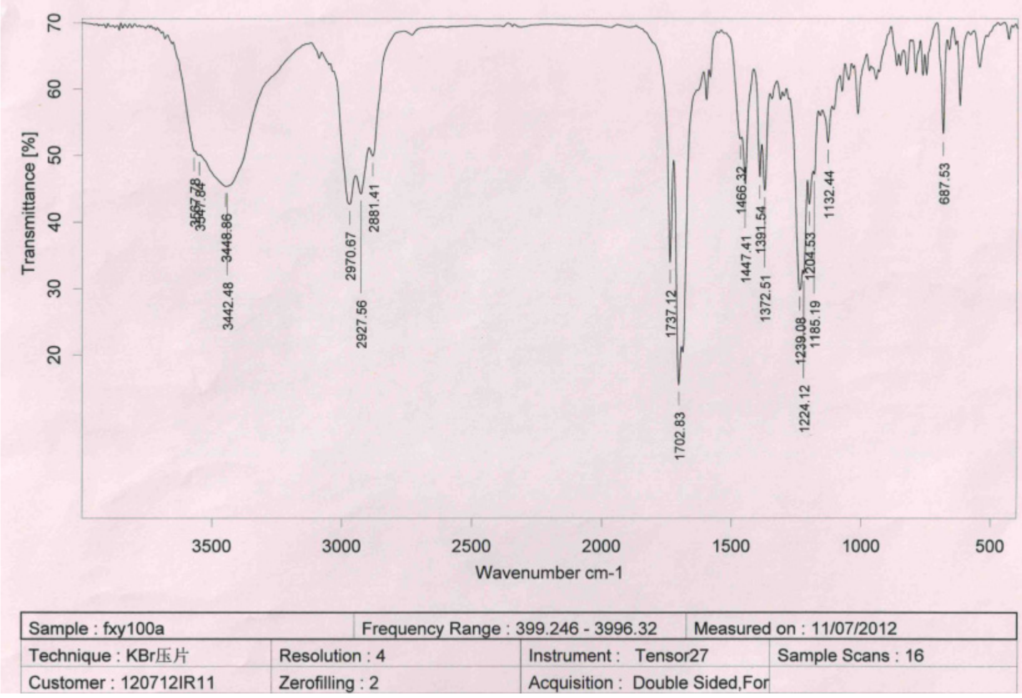

S10. UV spectrum of **1** in MeOH

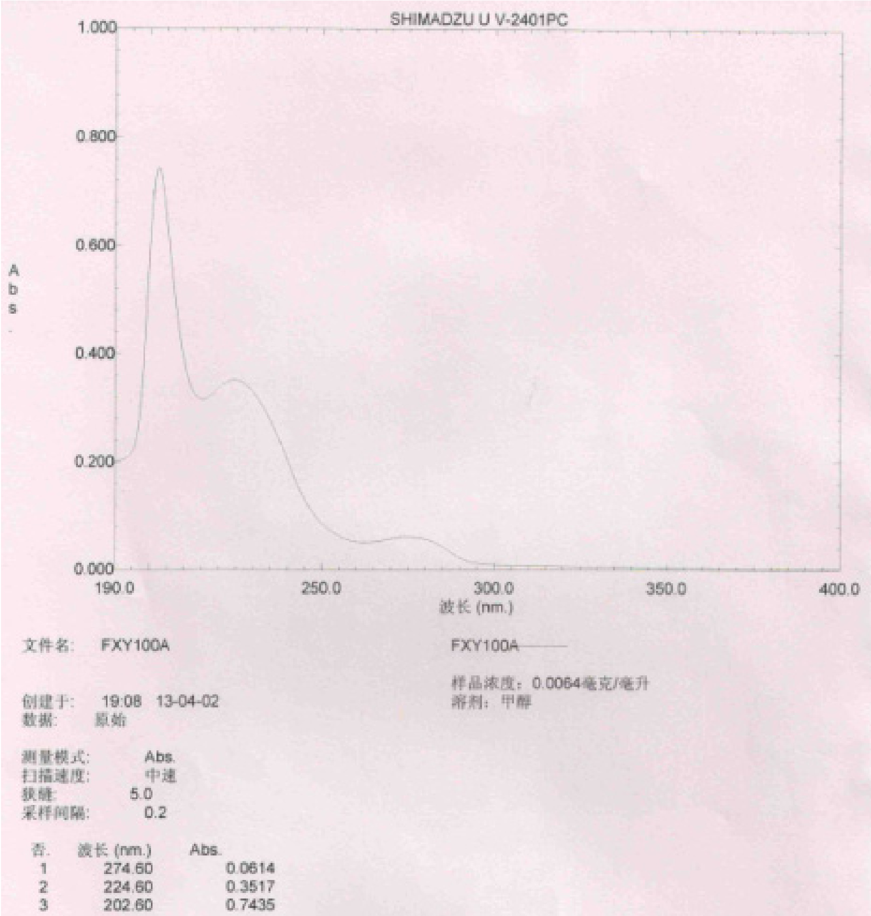

S11. ^{1}H NMR spectrum of **2** (acetone-d_6, 600 MHz)

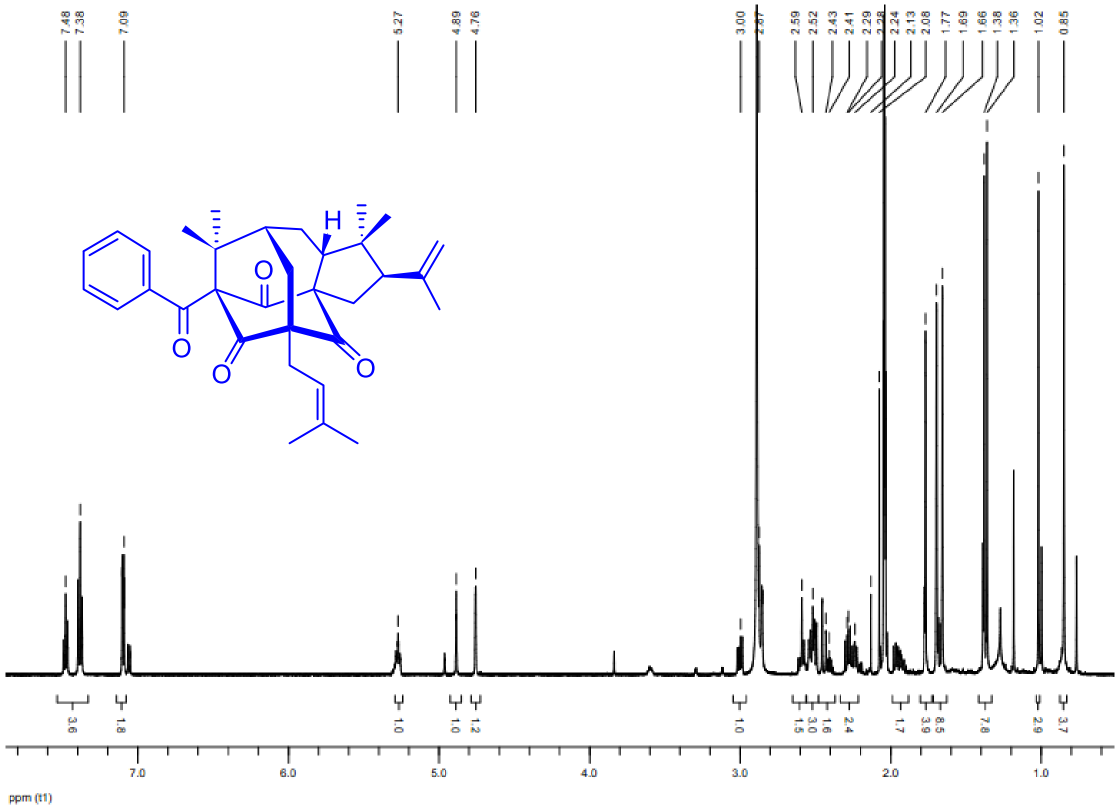

S12. ^{13}C NMR spectrum of **2** (acetone-d_6, 150 MHz)

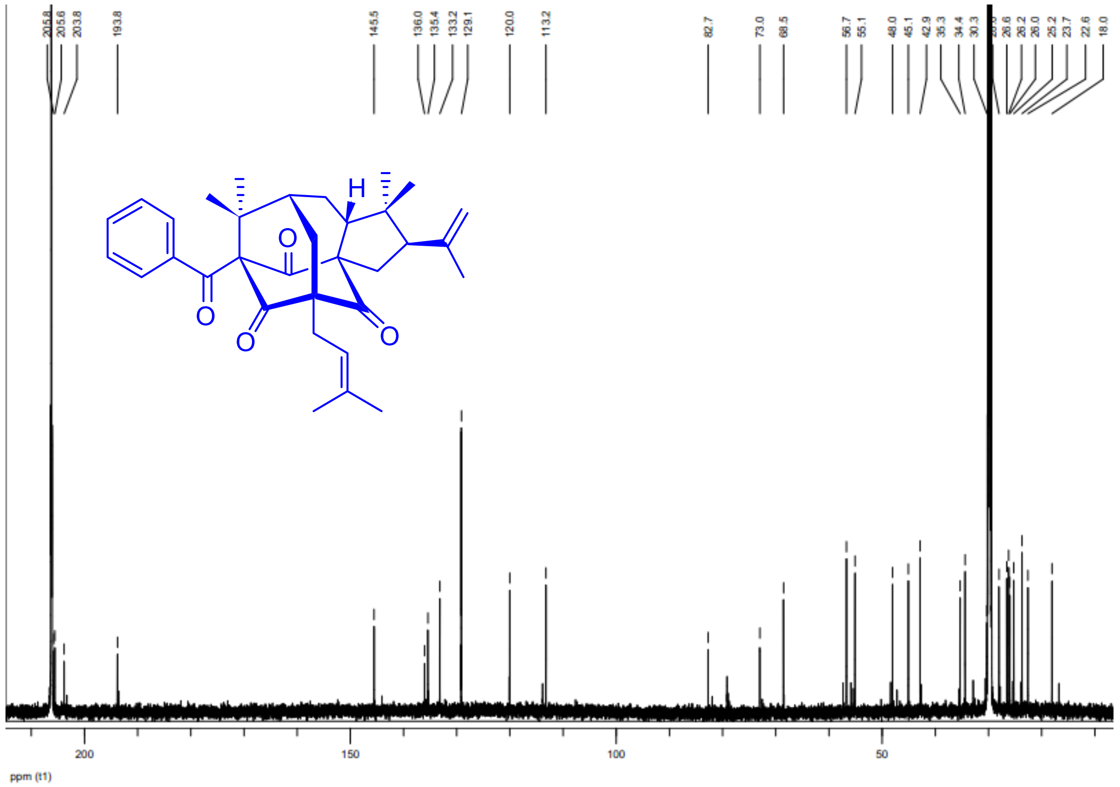

S13. HSQC spectrum of **2**

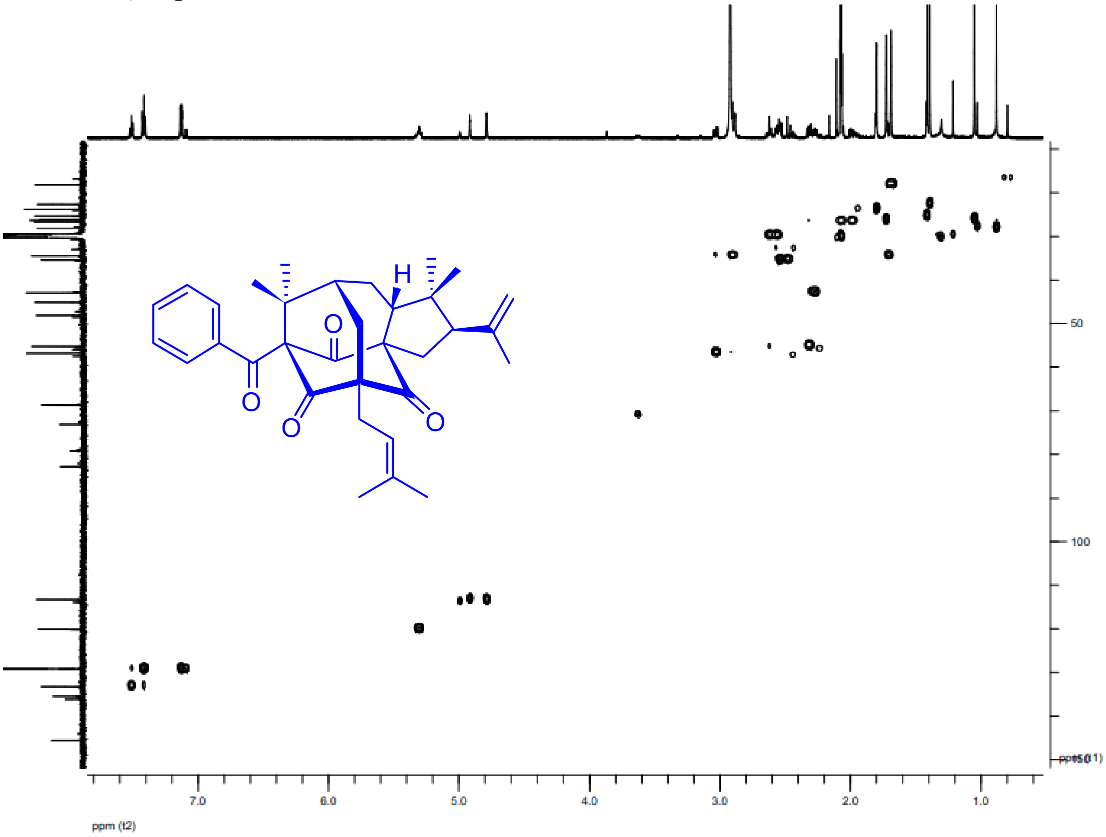

S14. HMBC spectrum of **2**

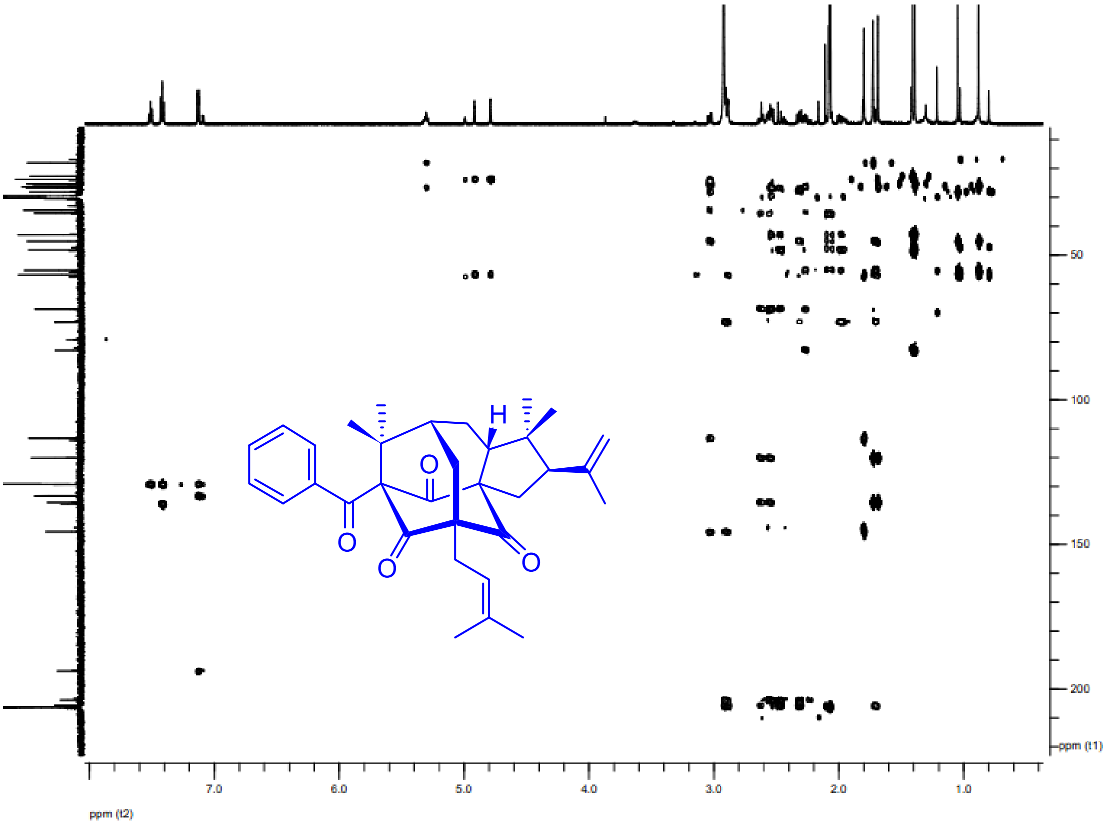

S15. ^{1}H-^{1}H COSY spectrum of **2**

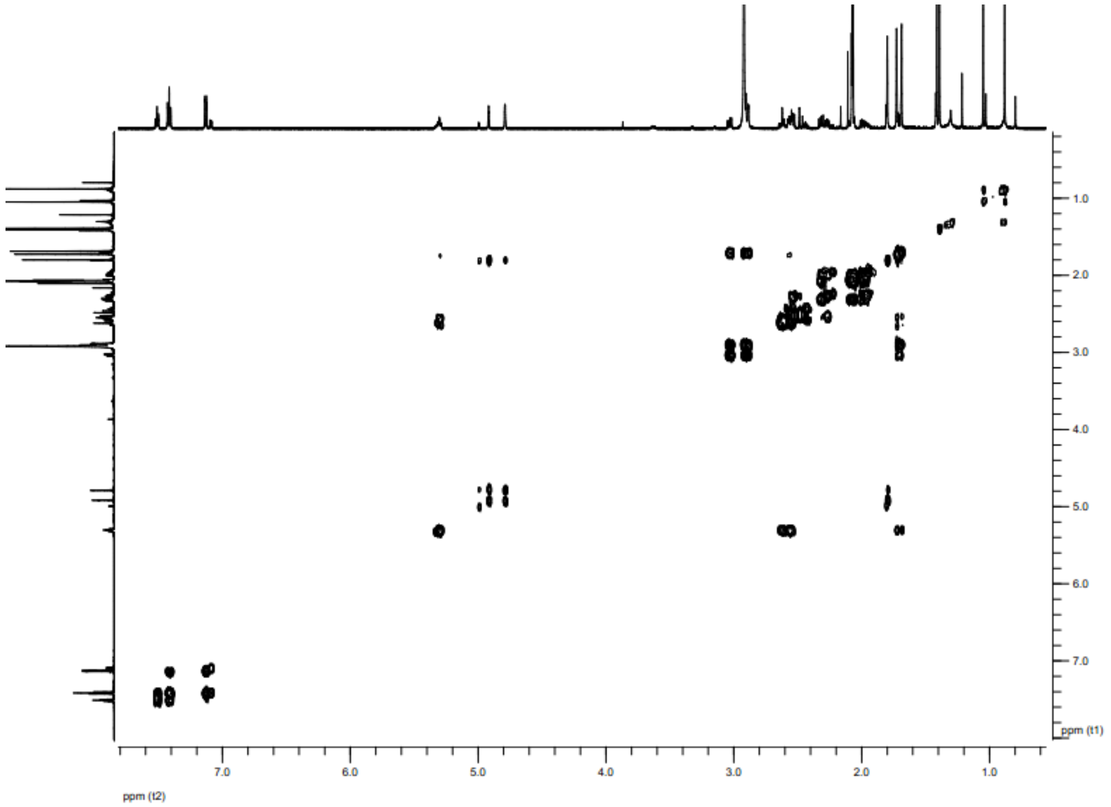

S16. ROESY spectrum of **2**

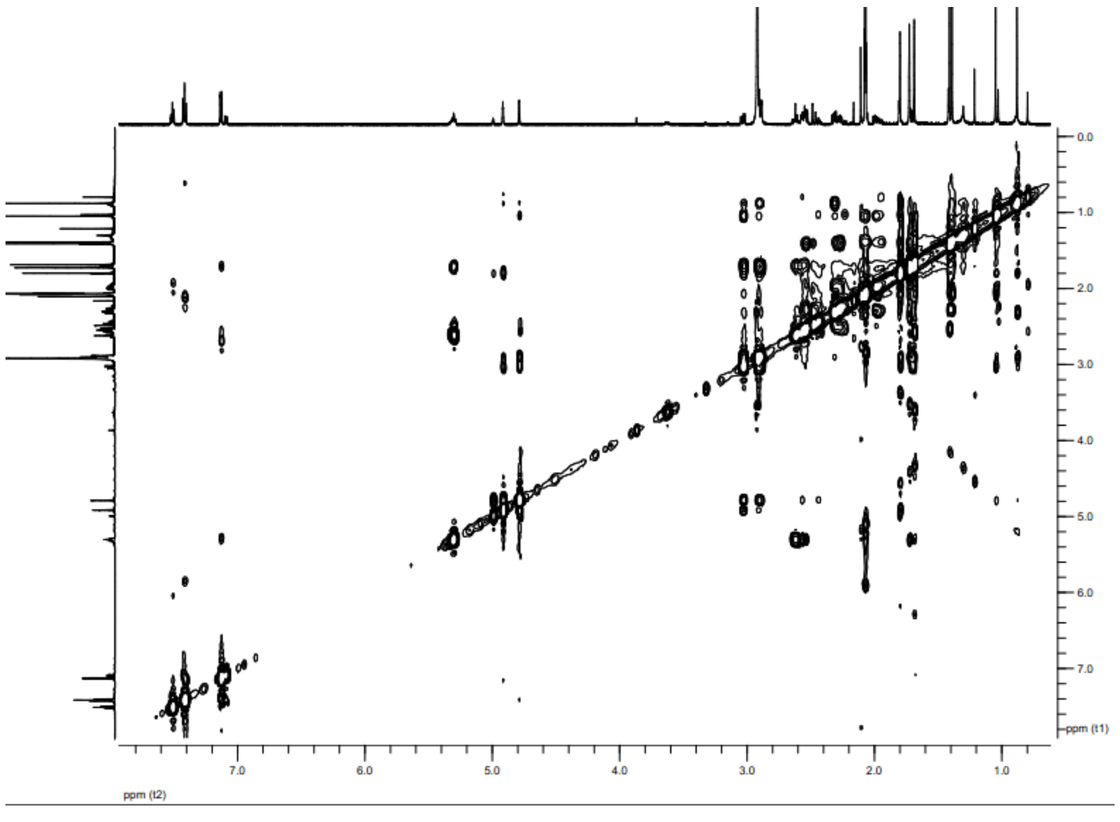

S17. ESI MS spectrum of **2**

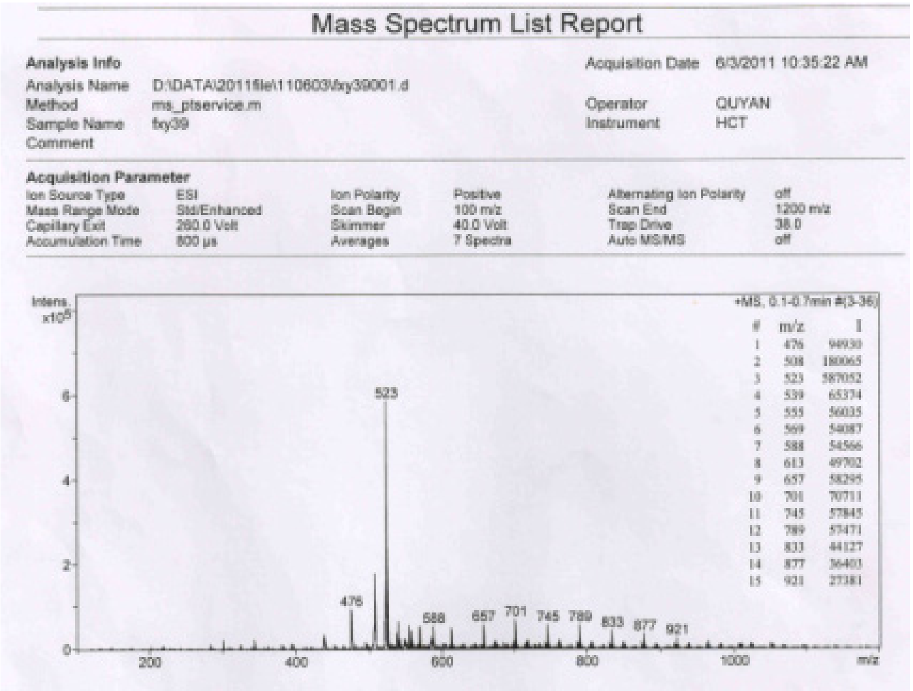

S18. HRESI MS spectrum of **2**

Acq. Date: Tuesday, June 07, 2011 Acq. Time: 11:47

Sample Name: 110607ESIA fxy39

Elemental composition calculator

Target m/z: +523.2811 amu
Tolerance: +10.0000 ppm
Result type: Elemental
Max num of results: 1000
Min DBE: -10.0000 Max DBE: +60.0000
Electron state: OddAndEven
Num of charges: 0
Add water: N/A
Add proton: N/A
File Name: 110607ESIA fxy39.wiff

	Elements	Min Number	Max Number
1	Br	0	0
2	C	0	200
3	Cl	0	0
4	F	0	0
5	H	0	400
6	K	0	0
7	N	0	0
8	Na	1	1
9	O	0	4
10	P	0	0

Acq. Date: Tuesday, June 07, 2011 Acq. Time: 11:47

Sample Name: 110607ESIA fxy39

	Elements	Min Number	Max Number
11	Pt	0	0
12	S	0	0
13	Si	0	0

	Formula	Calculated m/z (amu)	mDa Error	PPM Error	DBE
1	C33 H40 O4 Na	523.2824	-1.3298	-2.5413	13.5

S19. IR (KBr disk) spectrum of **2**

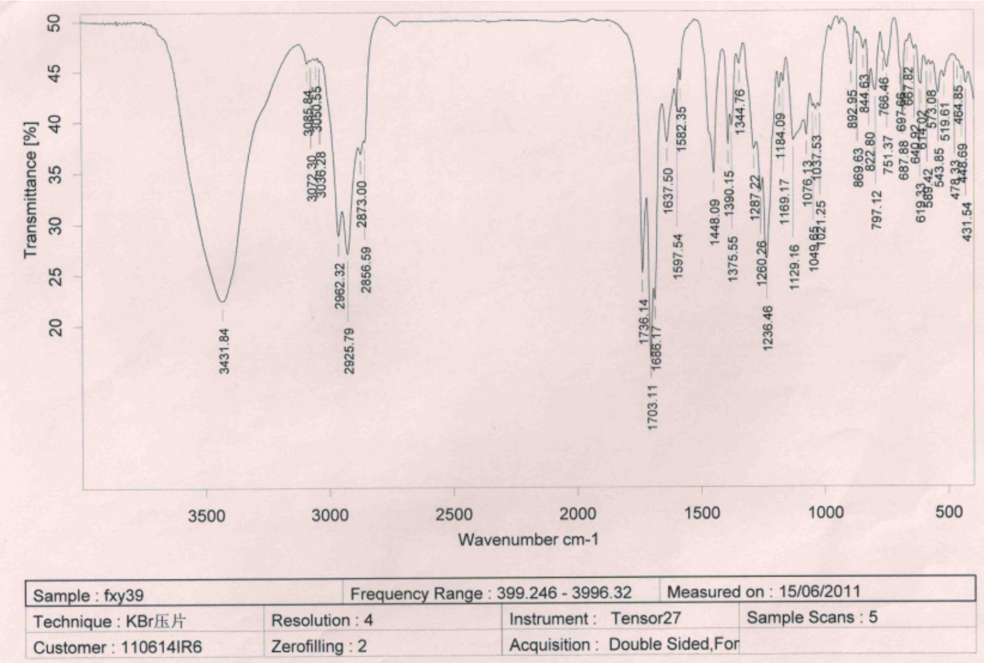

S20. UV spectrum of **2** in MeOH

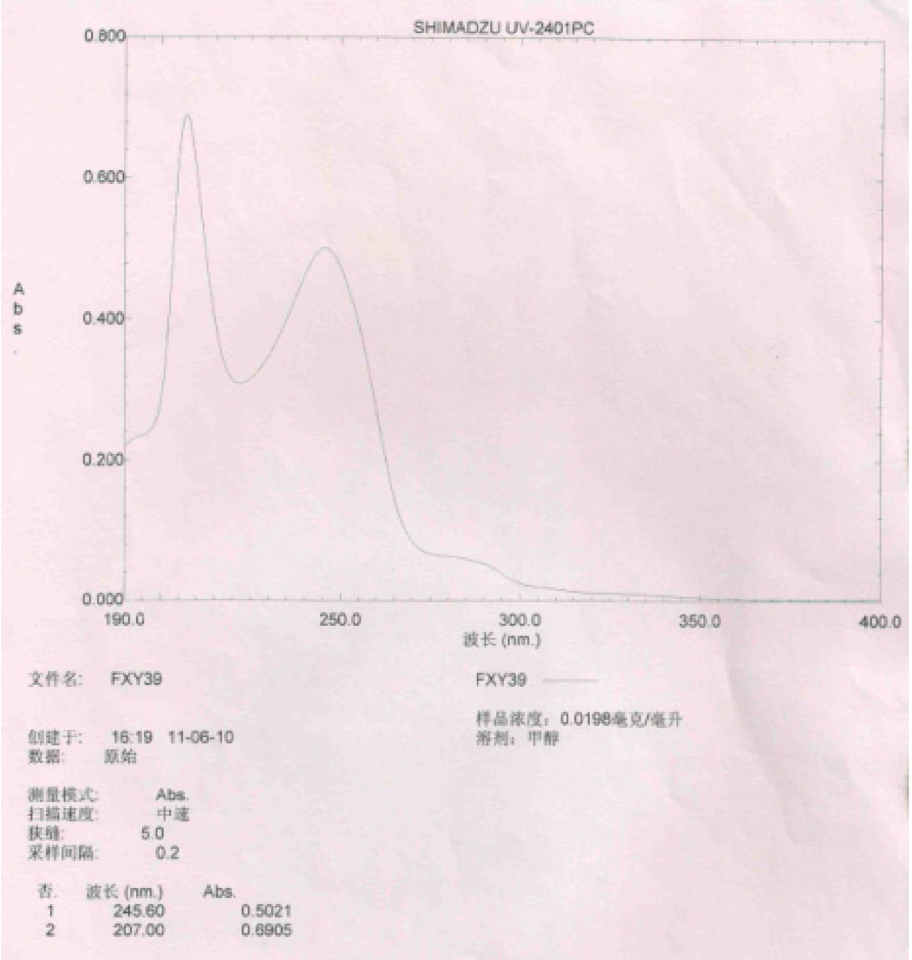

S21. ^{1}H NMR spectrum of **3** (acetone-d_6, 600 MHz)

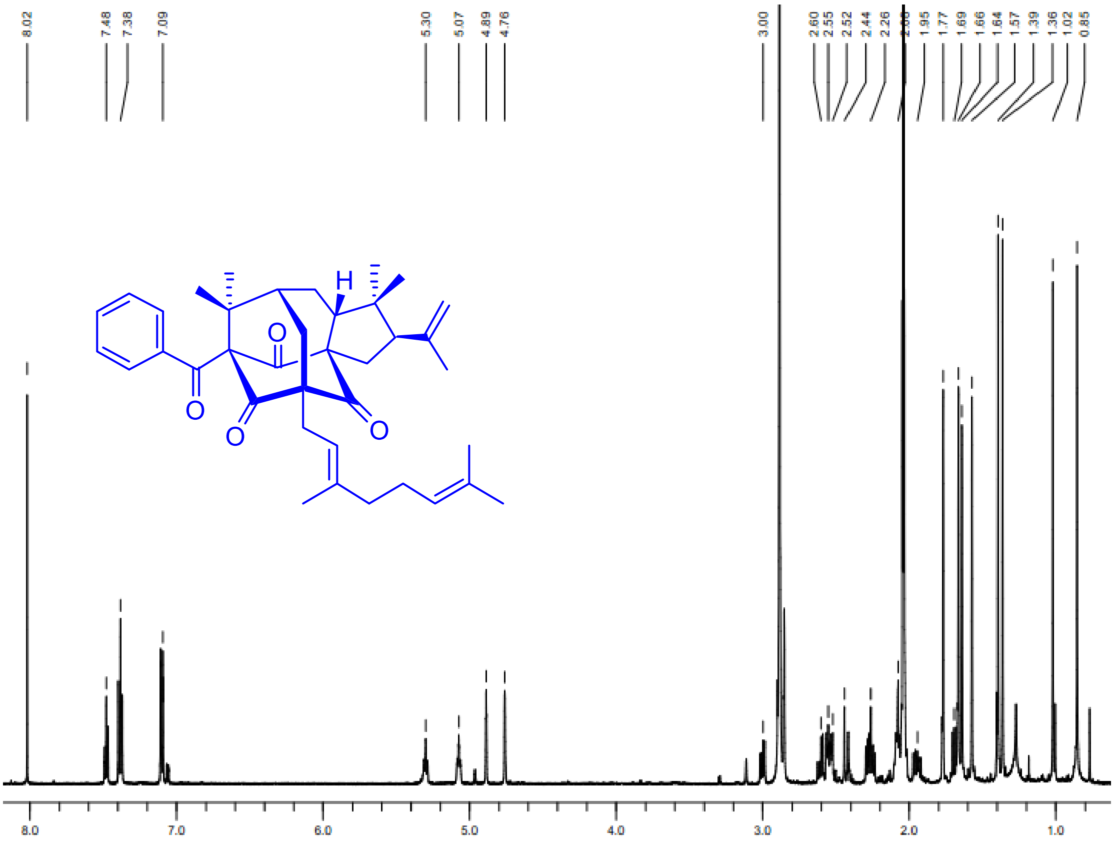

S22. ^{13}C NMR spectrum of **3** (acetone-d_6, 150 MHz)

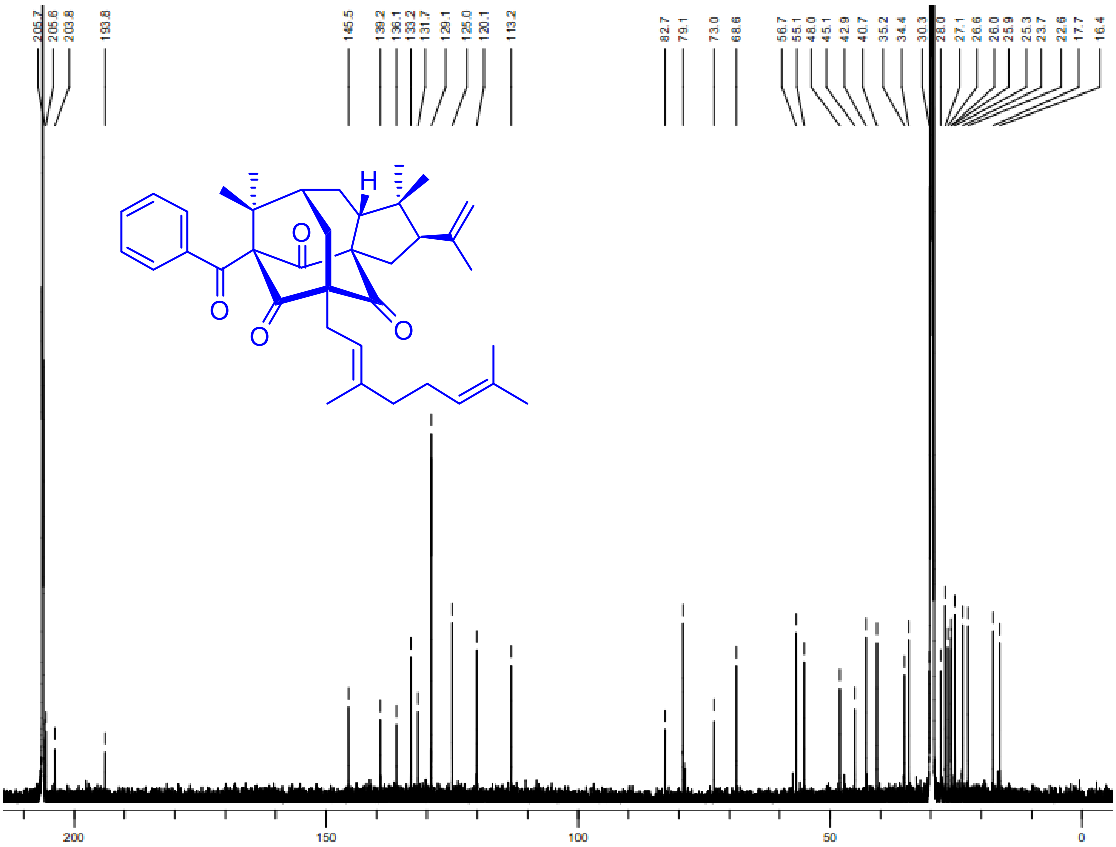

S23. HSQC spectrum of **3**

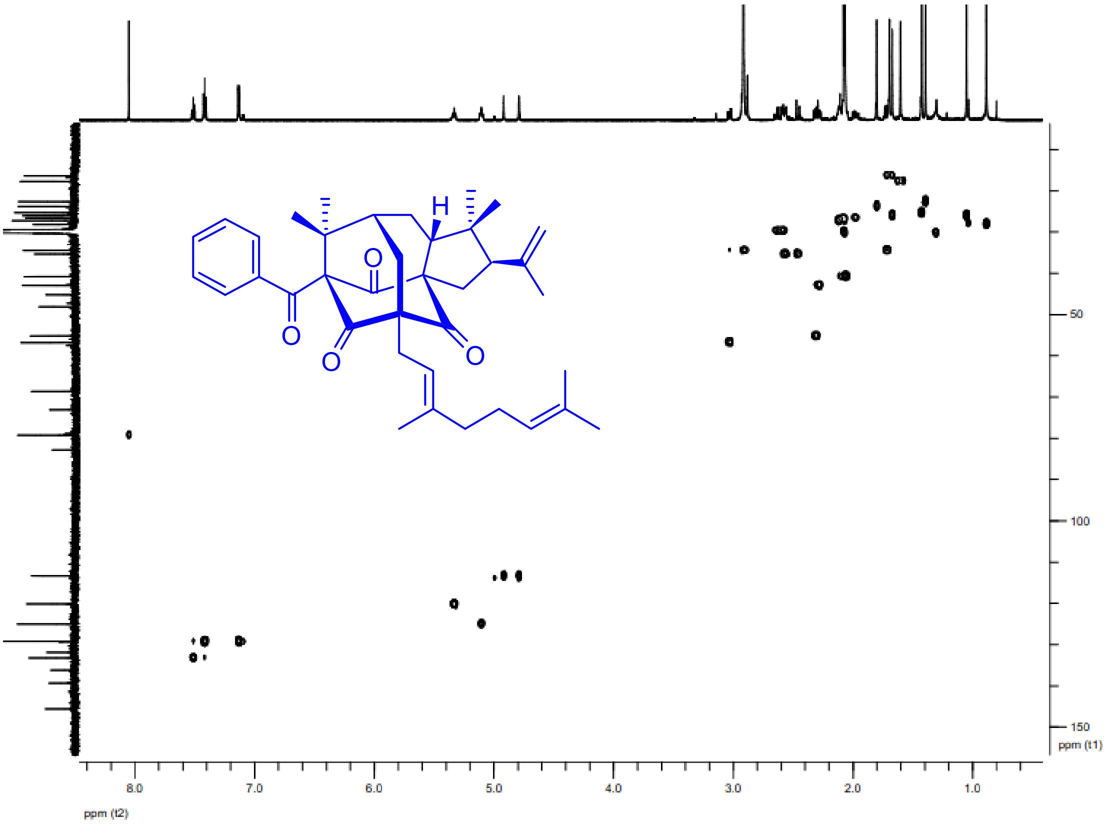

S24. HMBC spectrum of **3**

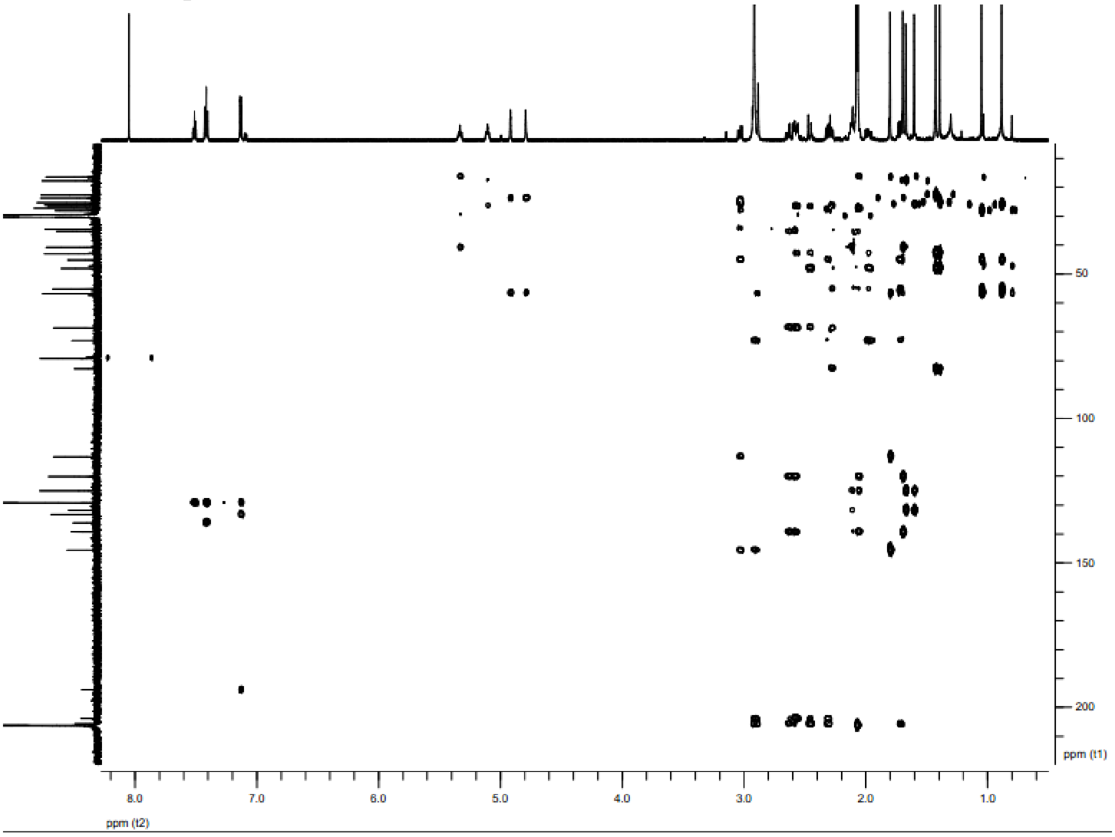

S25. ^{1}H-^{1}H COSY spectrum of **3**

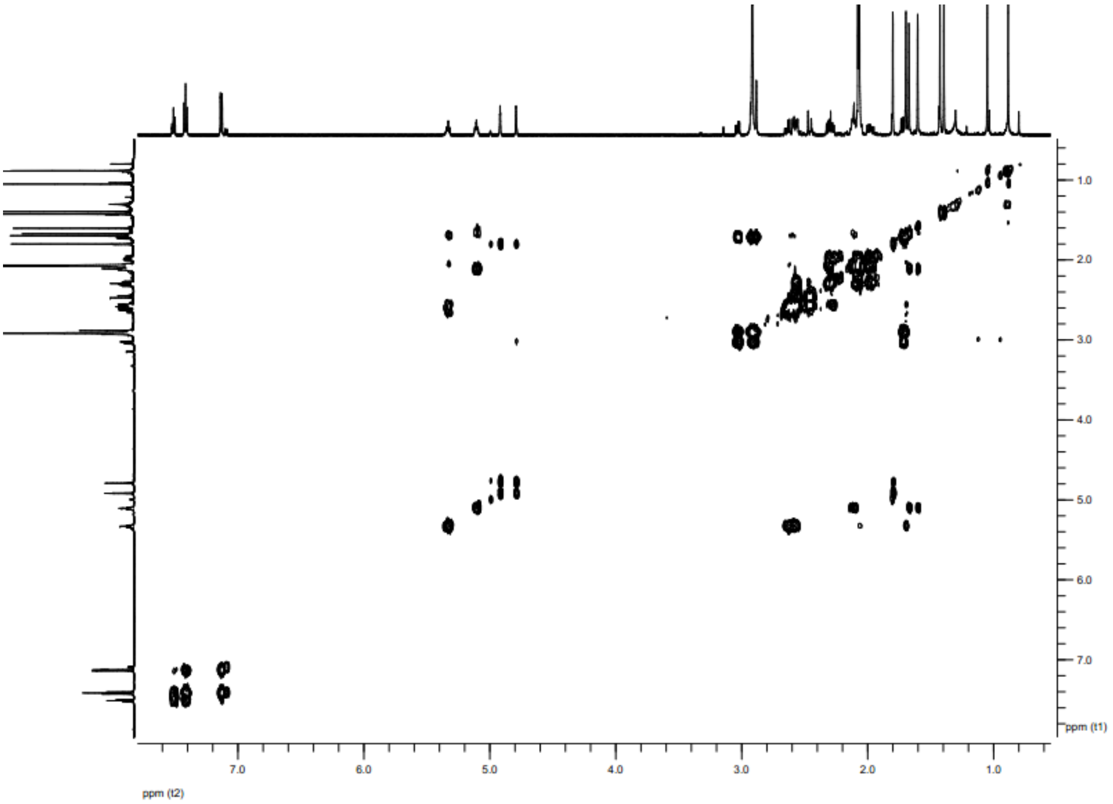

S26. ROESY spectrum of **3**

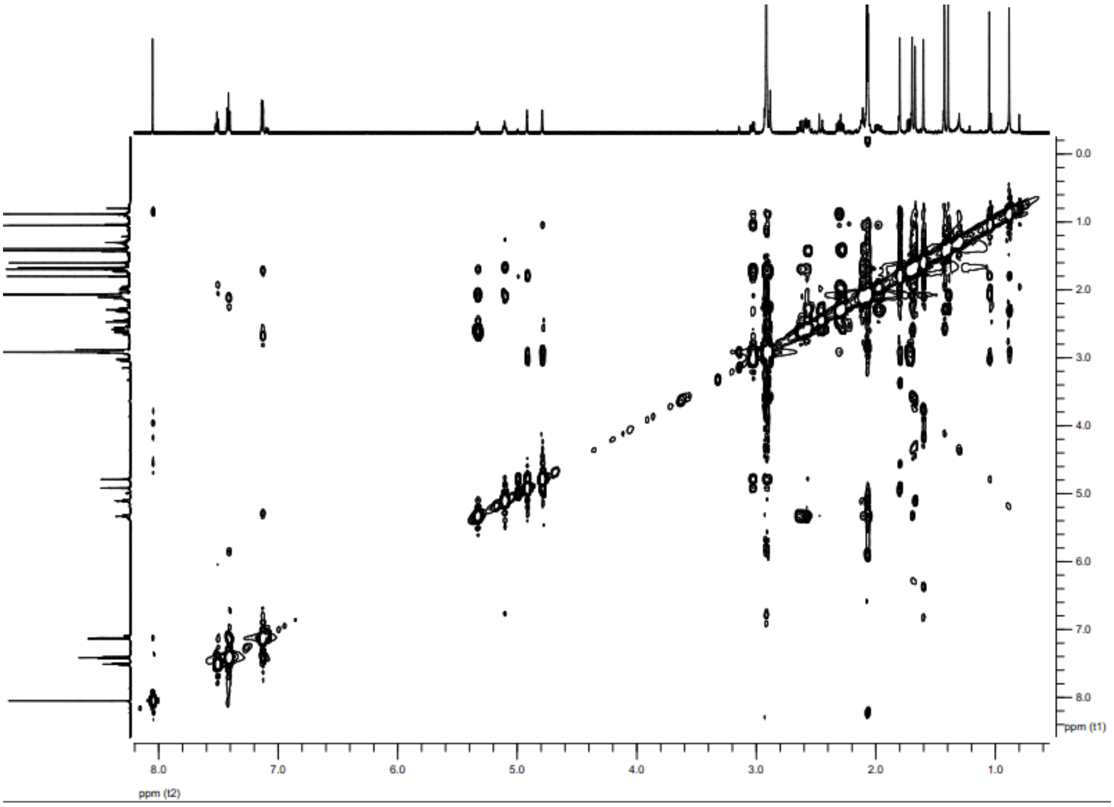

S27. ESI MS spectrum of **3**

Mass Spectrum List Report

Analysis Info

Analysis Name	D:\DATA\2011file\110603\fxy16.d	Acquisition Date	6/3/2011 9:49:08 AM
Method	ms_ptservice.m	Operator	QUYAN
Sample Name	fxy16	Instrument	HCT
Comment			

Acquisition Parameter

Ion Source Type	ESI	Ion Polarity	Positive	Alternating Ion Polarity	off
Mass Range Mode	Std/Enhanced	Scan Begin	100 m/z	Scan End	1300 m/z
Capillary Exit	200.0 Volt	Skimmer	40.0 Volt	Trap Drive	72.3
Accumulation Time	500 µs	Averages	7 Spectra	Auto MS/MS	off

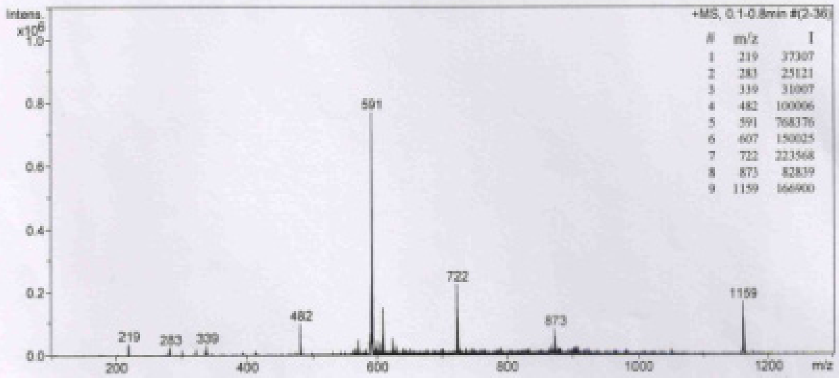

S18. HREI MS spectrum of **3**

Acq. Date: Tuesday, June 07, 2011 Acq. Time: 11:33

Sample Name: 110607ESIA fxy16

Elemental composition calculator

Target m/z: +591.3446 amu
Tolerance: +10.0000 ppm
Result type: Elemental
Max num of results: 1000
Min DBE: -10.0000 Max DBE: +60.0000
Electron state: OddAndEven
Num of charges: 0
Add water: N/A
Add proton: N/A
File Name: 110607ESIA fxy16.wiff

	Elements	Min Number	Max Number
1	Br	0	0
2	C	0	200
3	Cl	0	0
4	F	0	0
5	H	0	400
6	K	0	0
7	N	0	0
8	Na	1	1
9	O	0	4
10	P	0	0

Acq. Date: Tuesday, June 07, 2011 Acq. Time: 11:33

Sample Name: 110607ESIA fxy16

	Elements	Min Number	Max Number
11	Pt	0	0
12	S	0	0
13	Si	0	0

	Formula	Calculated m/z (amu)	mDa Error	PPM Error	DBE
1	C38 H48 O4 Na	591.3450	-0.4301	-0.7274	14.5

S19. IR (KBr disk) spectrum of **3**

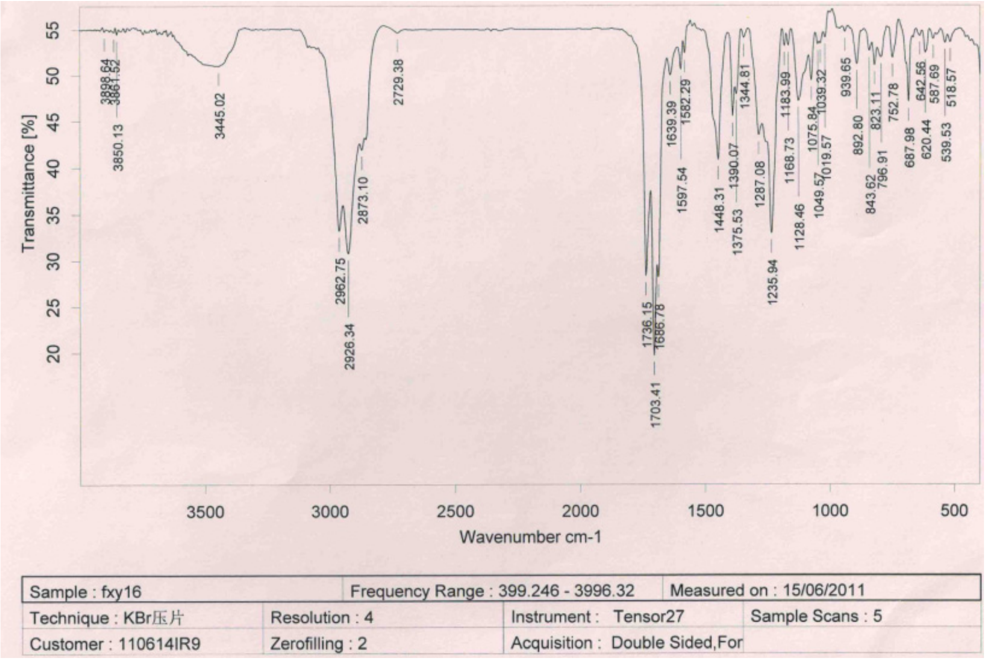

S20. UV spectrum of **3** in MeOH

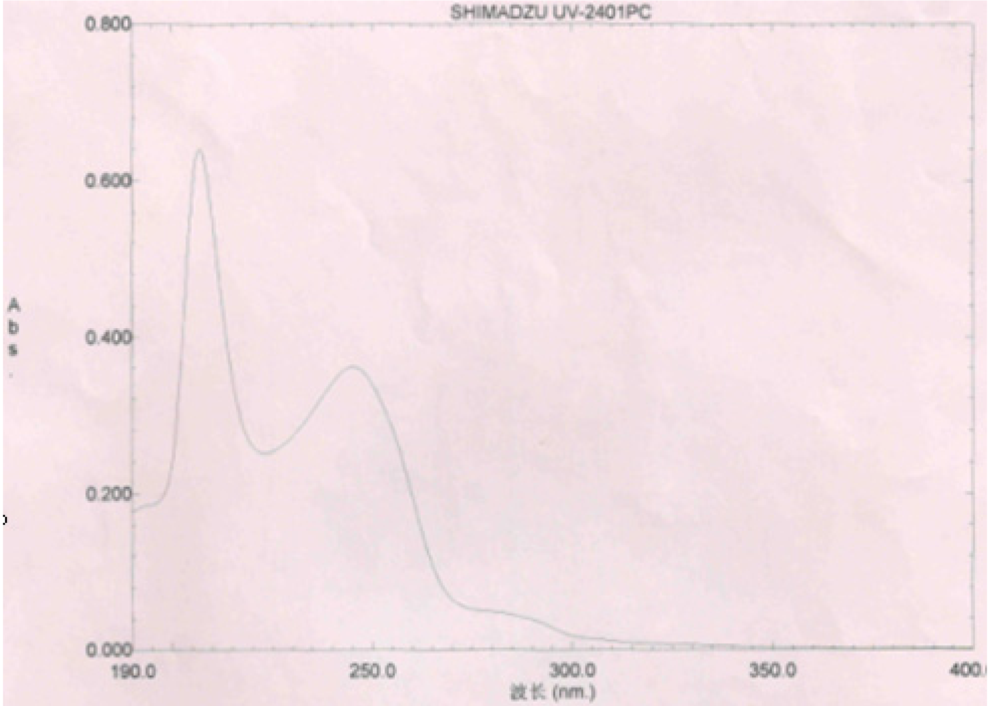

文件名: FXY16

FXY16 ———

样品浓度：0.0144毫克/毫升
溶剂：甲醇

创建于: 15:36 11-06-10
数据: 原始

测量模式: Abs.
扫描速度: 中速
狭缝: 5.0
采样间隔: 0.2

峰.	波长 (nm.)	Abs.
1	245.20	0.3613
2	207.00	0.6396
